# Supplementary material for: Cellular Consequences, Citrullination Substrates, and Antigenicity of Targeting PAD4 to Cell Surfaces
Source: ACS Cent Sci. 2026 Jul 21;12(8):1207–22. doi: 10.1021/acscentsci.6c00032 (PMC13523512; doi:10.1021/acscentsci.6c00032)
Supplement: Supplementary file 1 [file oc6c00032_si_003.pdf]

SUPPORTING INFORMATION

Cellular consequences, citrullination substrates, and antigenicity of targeting PAD4 to cell surfaces

Authors & Affiliations

Sophie Kong[1,2 ‡], Trenton M. Peters-Clarke[1 ‡], Corleone S. Delaveris[1,3], Paul Phojanakong[4], Veronica Steri[4], James A. Wells[1,4*]

[1]Department of Pharmaceutical Chemistry, University of California San Francisco, San Francisco, California, 94158, USA.

[2]Current address: Epi Biologics, San Mateo, CA 94403 USA.

[3]Current address: Inversion Therapeutics, San Francisco, California, 94158, USA.

[4]Preclinical Therapeutics Core, University of California San Francisco, San Francisco, California, 94158, USA.

[5]Department of Cellular & Molecular Pharmacology, University of California San Francisco, San Francisco, California, 94158, USA.

‡ Authors contributed equally

**Corresponding Author: jim.wells@ucsf.edu*

Figure S1. Schematic of transwell migration assay S2

Table S1. Top citrullinated protein hits identified from EMT6 proteomic analysis S3

Table S2. Top citrullinated protein hits identified from intact SKBR3 proteomic analysis S4

Figure S2. Tumor volume measured over time of subcutaneously implanted EMT6 tumors following vaccination S5

Figure S3. Breakdown of scan types within HCD-pd-EThcD for citrullination profiling of EMT6 lysate S6

Figure S4. Gene ontology enrichment for citrullinated proteins targeted by ZHER2-PAD4 within EMT6 lysate S7

Figure S5. Gene ontology enrichment for citrullinated proteins targeted by ZHER2-PAD4 within the surfaceome of SKBR3 cells S8

Figure S6. Representative incucyte images of EMT6 cells treated with PAD4 or controls S9

Figure S7. Treatment of EMT6 cells with active PAD4 changes cell morphology, growth rates, viability, and migration. S10

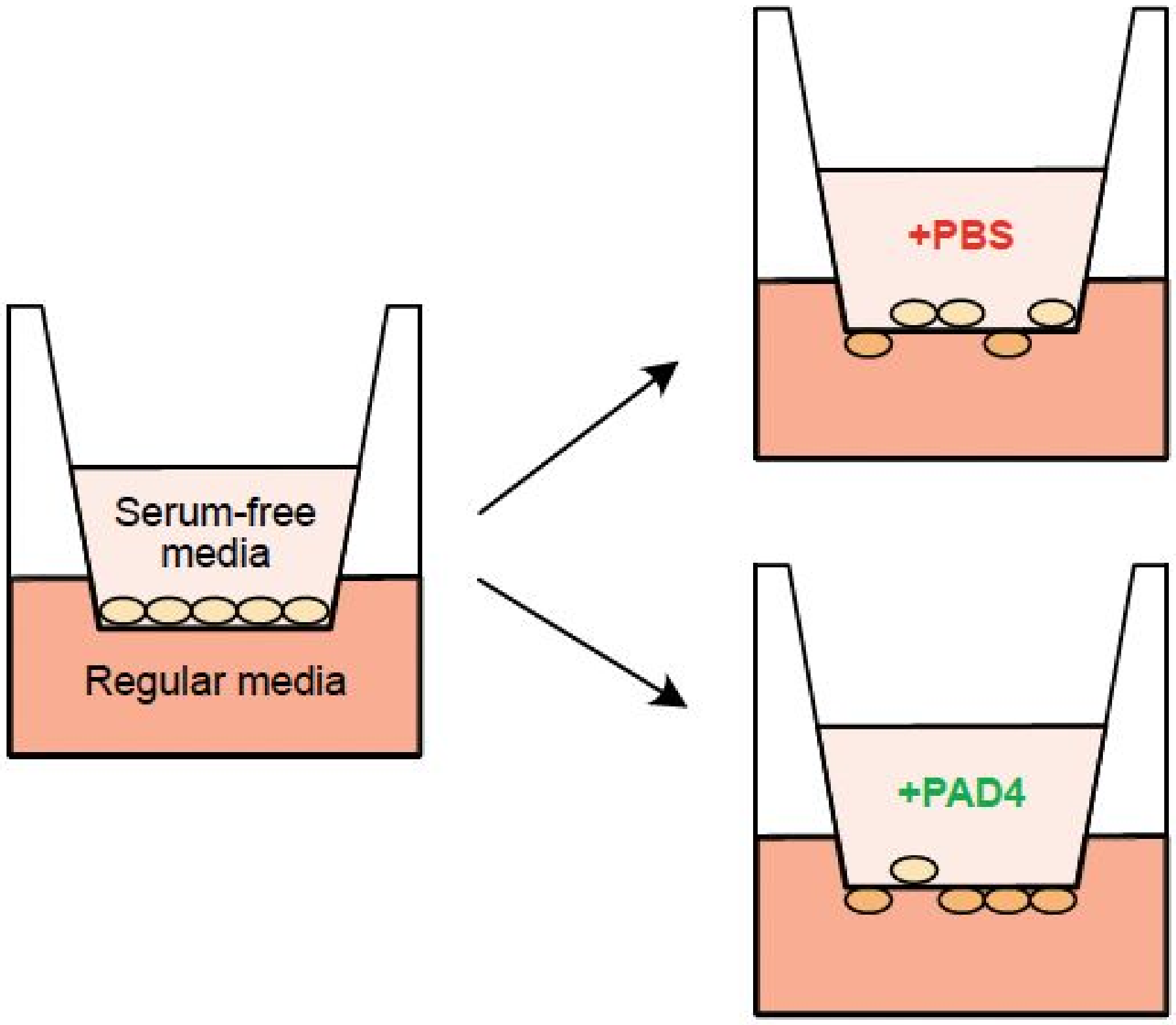

Supp Fig. S1. Schematic of transwell migration assay measuring the count of cells migrating from serum-free media (upper chamber) into the regular media (lower chamber).

PAD4	ERBB2	Erb-b2 receptor tyrosine kinase 2	Cytosol/membrane
	Tnks1bp1	Tankyrase 1 binding protein 1	Cytosol/membrane
	Pdap1	PDGFA interacting protein 1	Cytosol/membrane
	Rcc2	Regulator of chromosome condensation 2	Nuclear
	Ccdc124	Coiled-coil domain containing 124	Cytosol/membrane
	Dpysl2	Dihydropyrimidinase like 2	Cytosol/membrane
	Cavin1	Caveolae associated protein 1	Cytosol/membrane
	Rpsa	Ribosomal protein SA	Cytosol/membrane
	Cttn	Cortactin	Cytosol/membrane

ZHER2-PAD4	ERBB2	Erb-b2 receptor tyrosine kinase 2	Cytosol/membrane
	Aip	Aryl hydrocarbon receptor interacting protein	Cytosol
	Nedd4	NEDD4 E3 ubiquitin protein ligase	Cytosol
	Tnks1bp1	Tankyrase 1 binding protein 1	Cytosol/membrane
	Pdap1	PDGFA interacting protein 1	Cytosol/membrane
	Atp2a2	ATPase sarcoplasmic/endoplasmic reticulum Ca2+ transporting 2	Cytosol
	Cttn	Cortactin	Cytosol/membrane
	Tfrc	Transferrin receptor	Cytosol/membrane/endosomes/lysosomes
	Nisch	Nischarin	Nucleoplasm/cytosol
	Eps8l2	EPS8 like 2	Cytosol
	Ccdc124	Coiled-coil domain containing 124	Cytosol/membrane

Supplemental Table S1. Top citrullinated protein hits identified from EMT6 lysate proteomic analysis and their canonical location.

membrane associated and organization

Gene	Protein Name	Site(s)
HSPA9	Heat shock 70 kDa protein 9	491
MYH9	Myosin-9	930, 1652
SEC24C	Protein transport protein Sec24C	613
SLC25A4	ADP/ATP translocase 1	259
TIMM50	Mitochondrial import inner membrane translocase	398
VAPA	VAMP-associated protein A	58, 62, 127
VAPB	VAMP-associated protein B and C	38, 120
SLC25A5	ADP/ATP translocase 2	259
CANX	Calnexin	406
RRBP1	Ribosome-binding protein 1	1324
SAR1A	GTP-binding protein SAR1a	107
TMED10	Transmembrane emp24 domain-containing protein 10	45
VDAC2	Voltage-dependent anion-selective channel protein 2	10

stress / oxidative / NETosis

Gene	Protein Name	Site(s)
EEF2	Elongation Factor 2	66, 368, 580
FKBP1A	FK506-binding protein 1A	19
HBA2	Hemoglobin subunit alpha-2	32, 93
HNRNPK	Heterogeneous nuclear ribonucleoprotein K	148
IMPDH2	Inosine-5'-monophasphate dehydrogenase 2	105
OXSR1	Serine/threonine-protein inase OXSR1	14
PADI4	Protein-arginine deiminase type 4	372, 374, 441, 484, 555
PARK7	Protein deglycase DJ-1	48
PCNA	Proliferating cell nuclear antigen	91, 149
PPIA	Peptidyl-prolyl cis-trans isomerase A	37, 69
PSMD1	26S proteasome non-ATPase regulatory subunit 1	684
RANBP9	Ran-binding protein 9	333
SSB	Lupus La protein	246
TARDBP	TAR DNA-binding protein 43	208
TATDN1	TatD DNase domain-containing protein 1	119
TKT	Transketolase	213
TNIP1	TNFAIP3-interacting protein 1	482
UBA1	Ubiquitin-activating enzyme E1	79

Supplemental Table S2. Membrane associated, membrane organization, and stress-associated citrullinated proteins and their localized sites identified from intact SKBR3 proteomic analysis.

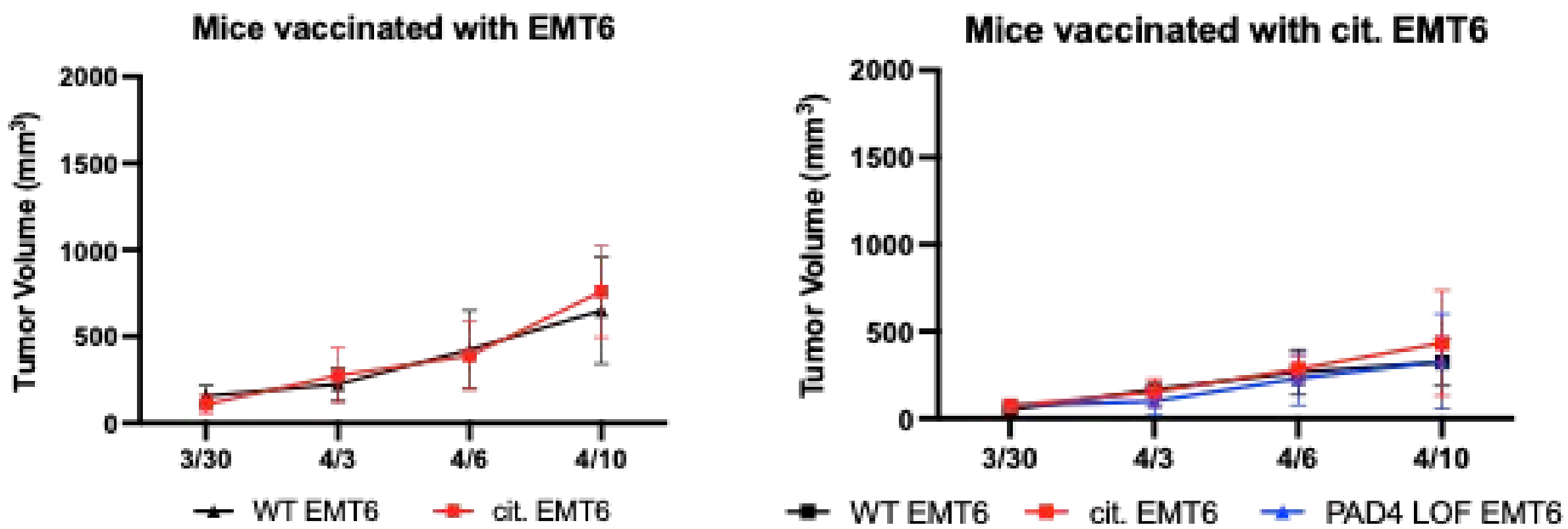

Fig. S2. Tumor volume measured over time of subcutaneously implanted EMT6 tumors following whole cell vaccination. Black plot corresponds WT EMT6 tumors implanted following vaccination, red corresponds to citrullinated tumors, and blue corresponds to tumor cells treated with inactive PAD4.

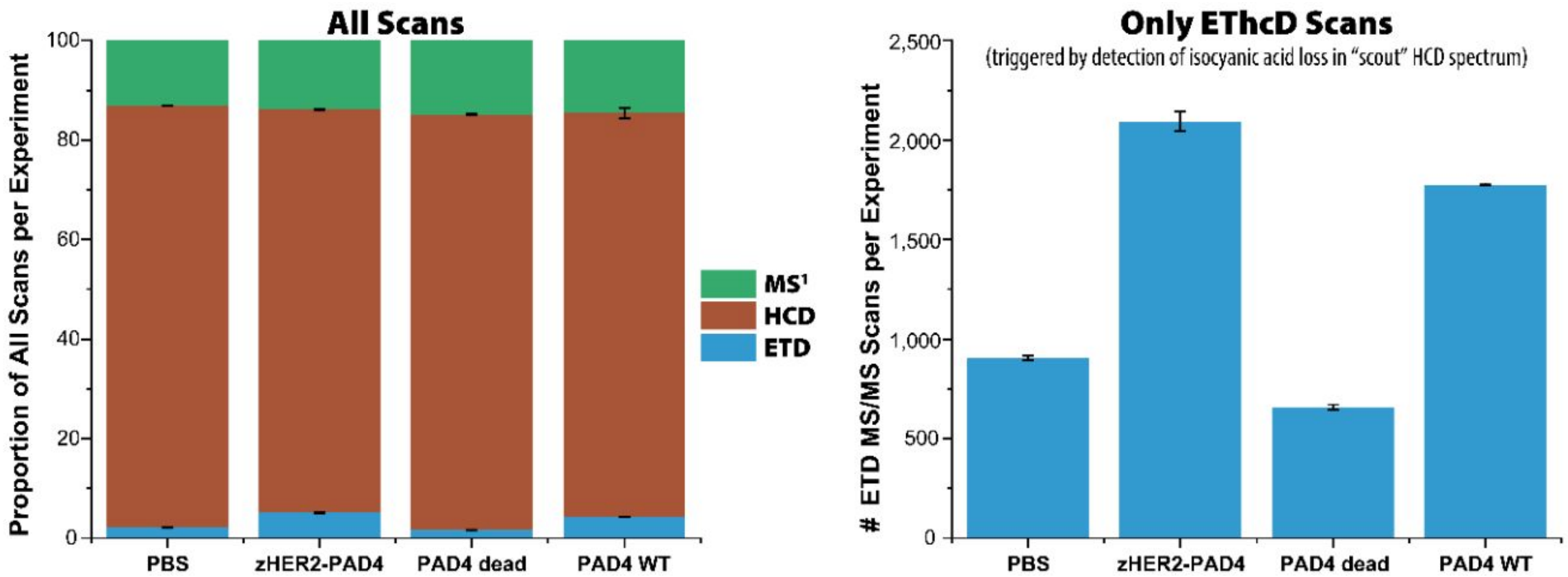

Fig S3. Breakdown of scan types within HCD-pd-EThcD intelligent data acquisition for citrullination profiling of EMT6 lysate. Triggering an EThcD scan necessitates longer scan times and slower duty cycles. Performing a quick HCD scan will release a labile isocyanate neutral loss from citrulline residues, an event that produces a unique diagnostic ion. Matching this mass shift (-43 Da) from the intact precursor (±10 ppm) in the scout HCD scan triggers a sequence informative and citrulline localizing EThcD scan. While all conditions produced some level of EThcD scans, the relative number of EThcD scans was highest for active PAD4 (WT and zHER2 targeted). The zHER2-PAD4 treatment of EMT6 lysate generated the highest number of triggered scans, over double the number of EThcD scans as the PBS and PAD4 LOF treatments. This indicates high enrichment of citrulline-containing peptides within the zHER2-PAD4 sample.

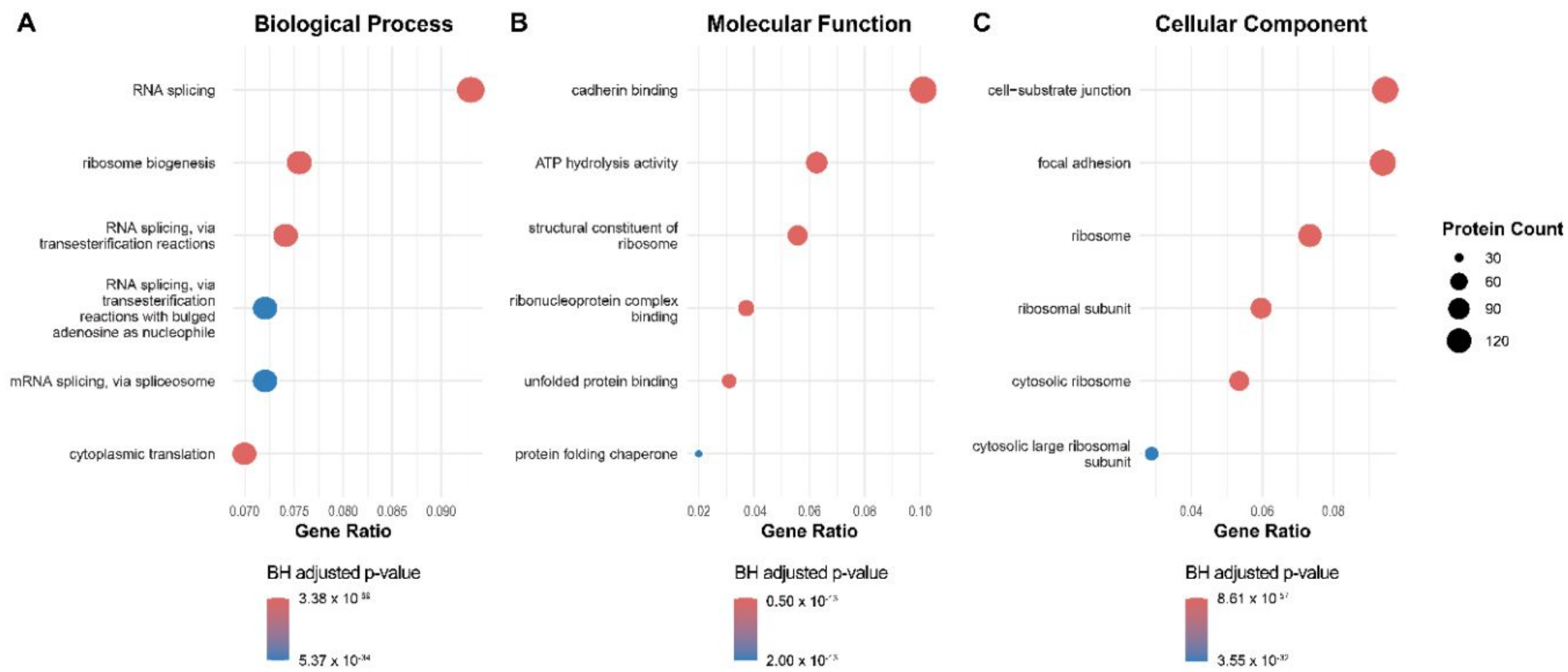

Fig S4. Gene ontology enrichment analysis for citrullinated proteins targeted by ZHER2-PAD4 within EMT6 lysate. Proteins were enriched according to their biological processes, molecular functions, and cellular components. Circle size represents the number of proteins in that gene ontology term, while the color of the circle represents the Benjamini-Hochberg (BH) corrected p-value. Gene ratio refers to the fraction of all citrullinated proteins by ZHER2-PAD4 that were categorized into the associated GO term.

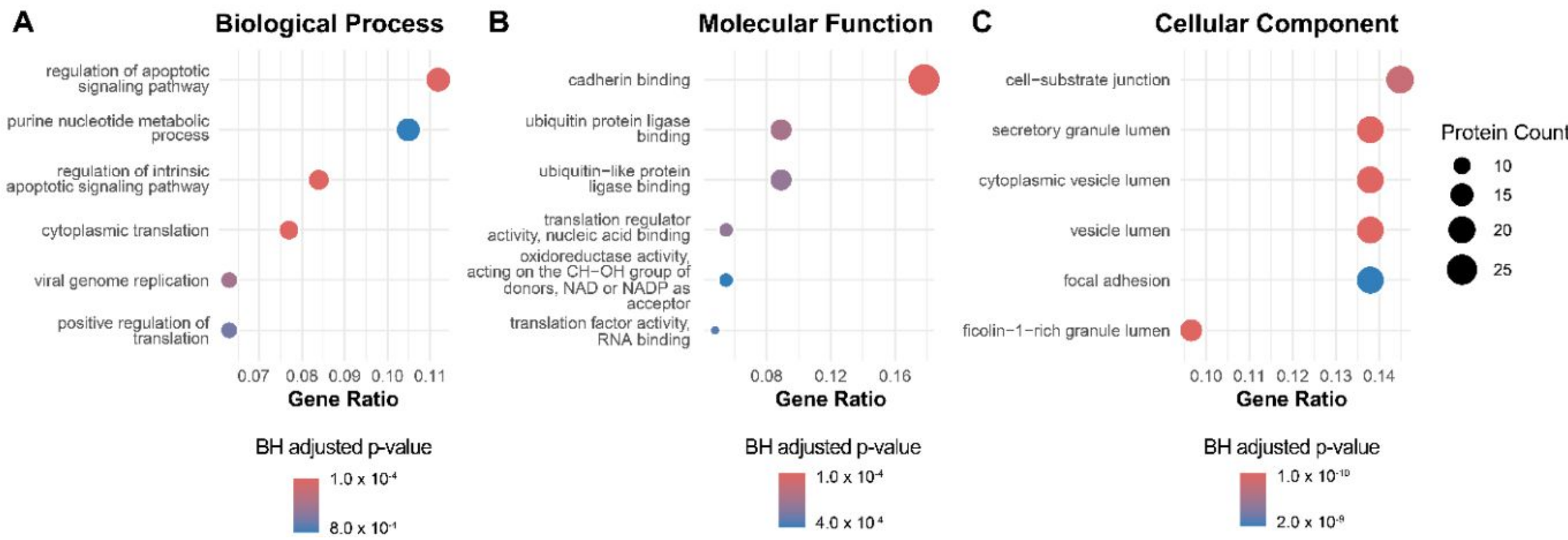

Fig S5. Gene ontology enrichment analysis for citrullinated proteins targeted by ZHER2-PAD4 within the surfaceome of SKBR3 cells. Proteins were enriched according to their biological processes, molecular functions, and cellular components. Circle size represents the number of proteins in that gene ontology term, while the color of the circle represents the Benjamini-Hochberg (BH) corrected p-value. Gene ratio refers to the fraction of all citrullinated proteins by ZHER2-PAD4 that were categorized into the associated GO term.

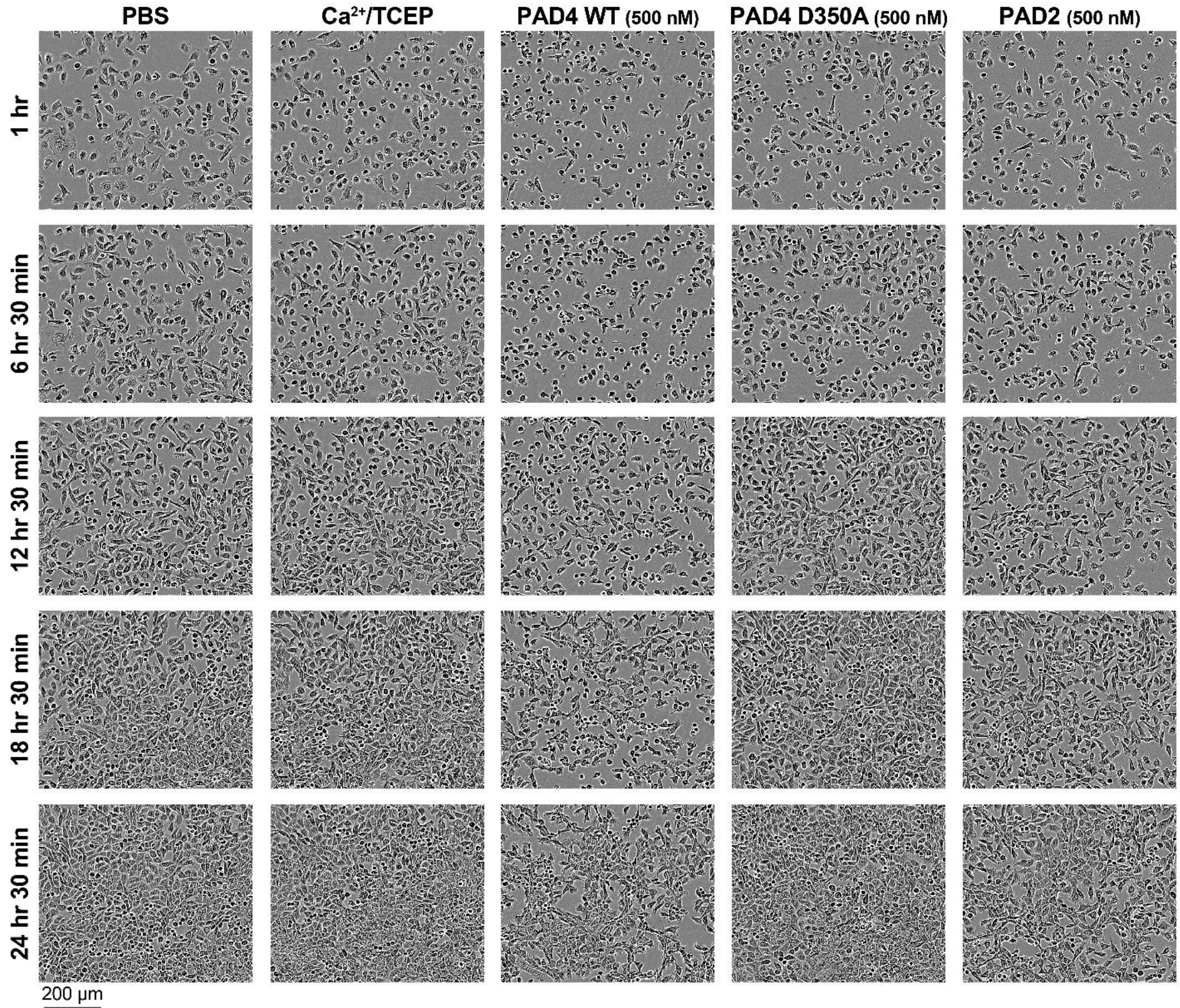

Fig. S6. Representative incucyte images of EMT6 cells treated with PAD4 or controls. Cells were treated for 24 hours and different parameters measured by incucyte. Brightfield images are shown for each of the five conditions at 1 hr through 24.5 hr following treatment.

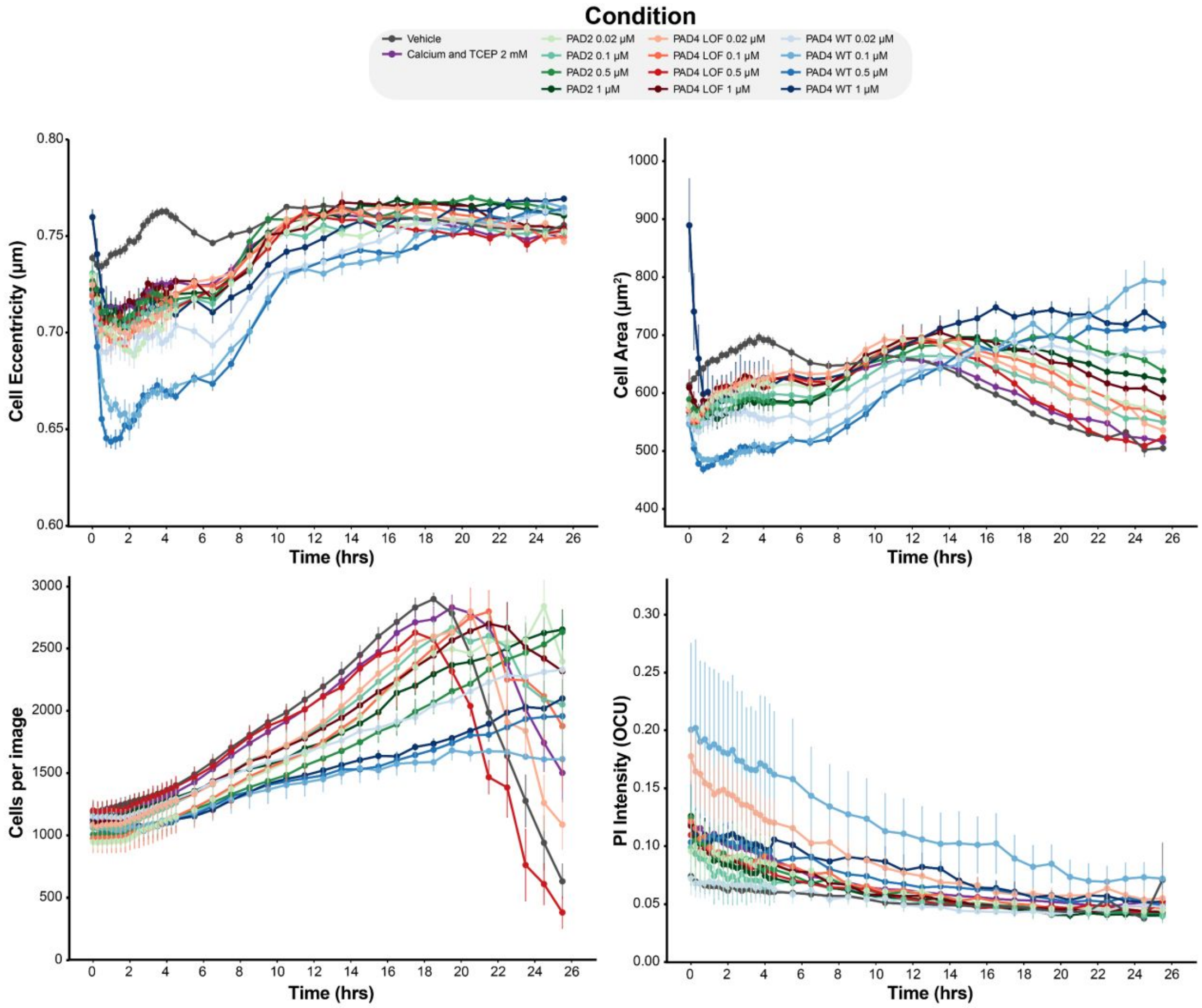

Supp Fig. S7. Treatment of EMT6 cells with active PAD4 changes cell morphology, growth rates, viability, and migration. Cells were treated for 24 hours and different parameters measured by incucyte. Cellular eccentricity defined as a less spherical cell shape, cell area, cell count, and cell viability measured by propidium iodine stain.
