# Supplementary material for: Cellular Consequences, Citrullination Substrates, and Antigenicity of Targeting PAD4 to Cell Surfaces
Source: ACS Cent Sci. 2026 Jul 21;12(8):1207–22. doi: 10.1021/acscentsci.6c00032 (PMC13523512; doi:10.1021/acscentsci.6c00032)
Supplement: Supplementary file 2 [file oc6c00032_si_005.pdf]

oc-2026-00032r.R1

Name: Peer Review Information for "Cellular consequences, citrullination substrates, and antigenicity of targeting PAD4 to cell surfaces"

First Round of Reviewer Comments

Reviewer: 1

Comments to the Author

This manuscript aims to understand the protein targets of extracellular protein arginine deiminase (PAD4) and the role of cell-surface citrullination in immunogenicity and cancer progression. The authors first demonstrate that treatment of EMT6 cells with PAD4 results in a change in morphology, generating more circular and less adherent cells. They then generated a ZHER2-PAD4 fusion protein where PAD4 is fused to the ZHER2 affibody, which would localize PAD4 at the surface of HER2-overexpressing cells. To identify protein targets of citrullination, the authors establish an HCD-product dependent-EThcD workflow. Negative control lysates (treated with DMSO or inactive PAD4) generated ~1000 citrullinated peptide identifications, whereas PAD4 or ZHER2-PAD4 samples afforded ~3000 citrullinated peptides. Lastly, EMT6 cells were citrullinated in vitro and used to immunize mice prior to implantation of EMT6 tumors. The resulting tumor take rate and the binding of mouse IgGs to citrullinated cells were measured.

Although the underlying premise and motivation for this study is of significant interest, the actual data that is presented falls short of providing significant insight into extracellular PAD function. The major concerns are outlined below:

1. The primary goal of this manuscript is to identify protein substrates of extracellular PADs, yet the main proteomic data provided is for cell lysates treated with exogenous PAD4. There are many studies out there that use a variety of methods to characterize PAD4 substrates in cell lysates and hundreds of citrullinated sites have already been identified. The main driving force for generating the HER2-PAD4 construct was to selectively look at

extracellular (and not intracellular) PAD targets, but these data are not presented. The authors state that SKBR3 cells were treated with PAD4, membranes isolated and analyzed, yet these proteomic data are not presented nor discussed in any detail.

2. More generally, the complete proteomic datasets were not provided making it difficult to judge the quality of the proteomic data.

3. The authors identify ~1000 citrullination sites with over 50% occupancy in non-PAD treated lysates, which is unusual because endogenous levels of PADs are typically low in most cell lines and endogenous citrullination stoichiometry is typically well below 50% for any given site of citrullination. More explanation needs to be provided to explain this high background.

4. Details regarding how 'citrulline localization confidence' and 'site occupancy' estimations were calculated need to be provided to again better judge the quality of the proteomic data.

5. The extensive discussion of HER2 citrullination sites seems extraneous to the main point of the manuscript which was to globally catalog cell-surface citrullination sites.

6. The measured differences in serum IgG binding to citrullinated cells (Fig 6 B, C and D) show a large degree of variability and low statistical significance. More data points would be helpful to more convincingly show that there is increased IgG binding.

7. Given the importance of the tumor take rate experiments, these data should be presented within the main text and not in the SI.

Reviewer: 2

Comments to the Author

This manuscript presents a technically ambitious and multifaceted study examining extracellular PAD4 biology, engineering a HER2-targeted PAD4 fusion construct (ZHER2–PAD4), and developing a neutral-loss–triggered HCD pd EThcD proteomics workflow for citrullination site identification. Using these tools, the authors map global and HER2 proximal citrullination sites and further investigate whether PAD4-mediated citrullination can act as a neoantigen source to stimulate humoral responses in a syngeneic mouse vaccination model. The work is innovative, and the proteomics pipeline represents a meaningful methodological advancement. The ZHER2–PAD4 fusion construct is well-designed and rigorously validated, and the authors successfully identify numerous endogenous and PAD4 dependent citrullination events. The manuscript addresses an important gap in understanding extracellular PAD4 biology and potential immunological consequences of citrullination.

However, significant conceptual, experimental, and interpretational issues remain. Several central claims—especially regarding proximity-based substrate identification, biological specificity of PAD4 activity, and conclusions drawn from the vaccination studies—are currently overstated or insufficiently supported. Logical gaps also arise from sudden transitions between major research components. Addressing these weaknesses would considerably strengthen the manuscript.

Major Concerns:

1. Conceptual inconsistency and logical gaps

A. Abrupt transitions between major components

The manuscript shifts rapidly from:

1) Phenotypic effects of exogenous PAD4 →
2) Engineering ZHER2–PAD4 →
3) Lysate-based global citrullination mapping →
4) Intact cell–specific HER2 citrullination →
5) Vaccine immunology studies.

The narrative lacks connective tissue explaining why each transition is made and how they inform one another, which leads to two partially disconnected storylines: a targeted PAD4 engineering/proteomics story, and a general PAD4 based cancer vaccine story.

The authors should add clearer rationale linking each experimental module and clarify whether the primary scientific goal is mechanistic dissection, method development, or translational immunology.

2. Problems with the use and interpretation of lysate based proximity studies

A. Lysates cannot support claims of proximity-induced substrate selection

Lysate experiments destroy spatial protein organization, permitting promiscuous solution phase citrullination. Multiple reviewers correctly point out that the claimed “proximal” substrates in Fig. 4 are not meaningful in the context of induced proximity.

The authors should clarify that: lysate proteomics measures enzyme preferences, not spatial interactions; only intact-cell SKBR3 experiments support proximity regulated citrullination.

B. Very high background citrullination in PBS and LOF controls

Detection of >1000 endogenous sites in controls raises concern about false positives or neutral-loss–triggered ID artifacts. The authors should validate a subset of endogenous hits with orthogonal methods or provide FDR benchmarking.

C. Missing essential negative control

PAD4 LOF was used instead of ZHER2–PAD4 LOF, despite the latter being available and more appropriate.

3. Inconsistencies in PAD4 binding and activity across assays

Several discrepancies must be clarified:

- WT PAD4 binds HER2+ cells by flow cytometry (Fig. 2C), yet shows nearly no citrullination by Western (Fig. 2E).
- SKBR3 intact-cell MS shows no WT PAD4 citrullination of HER2, contradicting earlier data.

The authors should discuss assay sensitivity, kinetics, and potential differences between biochemical and intact cell environments.

4. Issues with Figure 1 and biological interpretations

- No images are provided to support quantified morphology metrics.
- N values for all panels (A–D) are missing.
- Changing cell line (EMT6 → Patu8988t) for migration studies lacks justification and biological rationale.

Recommendation: The authors should add representative images, replicate numbers, reasoning for cell-line choice, and discussion connecting morphology to specific citrullinated substrates.

5. Mass spectrometry workflow: missing performance metrics

- No assessment of neutral loss triggering efficiency.
- No comparison with standard ETD workflows in complex samples.
- No discussion of potential false negatives.

The authors should include benchmarking or limitations discussion.

6. HER2 citrullination analyses: conflicting statements and missing data

- Authors state only ZHER2–PAD4 produces extracellular citrullination on HER2, but text and figures appear partially inconsistent.
- Quantitative summary of SKBR3 citrullinated peptide counts is missing.

7. Immunization study design issues

A. Missing pre challenge serum timepoint

Without Week 0 serology, it is impossible to isolate vaccine induced immunity from tumor induced recall responses.

B. Lack of negative target controls The authors claim antibodies target citrullinated epitopes, but binding to non citrullinated cells is not shown.

C. Lack of T-cell functional readouts

The manuscript claims immunological relevance but provides only IgG binding data.

The authors should either temper claims or include additional functional immunology data.

Minor Issues and Required Corrections

(Condensed for clarity—full details reflected directly from reviewers)

- Ensure consistent terminology (e.g., EMT6 vs EMT 6).
- Provide gating strategies for all flow cytometry experiments.
- Improve clarity of figure labels, especially in Fig. 5.
- Cite prior publications where needed (e.g., histone H3 citrullination sites).
- Add methodological details on data normalization, missing value handling, heatmap scaling.

Overall, this manuscript has high potential for impact in chemical biology, proteomics, and immunology. The engineered ZHER2–PAD4 and the HCD pd EThcD workflow are valuable contributions that many groups will adopt. The proteomic dataset will serve as a foundational resource for the citrullination community. However, publication in ACS Central Science requires substantial conceptual clarification and strengthening of experimental interpretations. Addressing the issues above—especially proximity claims, methodological limitations, inconsistencies across assays, and vaccination study design—will greatly enhance rigor and coherence.

Author's Response to Peer Review Comments:

Dear Editor,

Thank you for the opportunity to revise our manuscript. We appreciate the enthusiasm and constructive comments from the Reviewers. We highlight the revisions made in response to the Reviewer comments, changes which we believe greatly improve the manuscript’s quality and place the results in a more cohesive narrative.

You mentioned several aspects that we should address in our revised manuscript, and we address them in the attached letter.

Dear Editor,

Thank you for the opportunity to revise our manuscript. We appreciate the enthusiasm and constructive comments from the Reviewers. We highlight the revisions made in response to the Reviewer comments, changes which we believe greatly improve the manuscript's quality and place the results in a more cohesive narrative.

You mentioned several aspects that we should address in our revised manuscript, and we address them below:

Formatting Needs:

Abstract: Please make sure the word count of your Abstract does not exceed 200 words.

We have adjusted the Abstract text to meet the 200 word maximum.

Ethics Statement: Your experimental section appears to detail the use of human or animal samples/participants. As per ACS's ethical guidelines section B15, "The American Chemical Society Publications rules and ethical guidelines provide mandatory standards of practice in experimental studies performed using biological samples obtained from animals or human subjects. Studies submitted for publication approval must present evidence that the described experimental activities have undergone local institutional review assessing safety and humane usage of study subject animals. In the case of human subjects, authors must also provide a statement that study samples were obtained through the informed consent of the donors, or in lieu of that evidence, by the authority of the institutional board that licensed the use of such material. Authors are requested to declare the identification or case number of institution approval as well as the name of the licensing committee in a statement placed in the section describing the studies' Material and Methods."

Please add a statement to your methods section detailing your ethics approval process for the use of animal/human participants. The statement should provide the identification or case number of institutional approval as well as the name of the licensing committee that approved the experiments. For research involving human participants/samples, please also include a statement detailing your informed consent protocol.

We include details of the experiments involving mice within the Methods:
"All animal procedures were approved by the University of California San Francisco (UCSF) Institutional
Animal Care and Use Committee (IACUC) (Protocol #AN206976), and complied with the NIH Guide for the Care and Use of Laboratory Animals. Mice were housed with ad libitum food and water on a 12-h light cycle at the UCSF Preclinical Therapeutics Core vivarium under pathogen-free, ambient temperature and humidity housing conditions." (page 27, line 559)

Graphics: If a figure has parts labeled (i.e. a, b, etc.), each part must be mentioned in the figure caption. Please see Figure 4, which is missing subpart D in the caption.

We have added a description of Figure 4D to the caption.

Graphics: Copyright permissions are required for any graphic that is reproduced or adapted from a source that is not published by the American Chemical Society. See, for example, Figures 3, 4, and 5. Please make sure that all graphics reproduced or adapted from another journal have the proper permission forms and credit lines in your revision. If the document list is extensive, you may consider compressing the files and uploading a ".zip" file "for Editors only". For more information on image permissions, see:
https://urldefense.com/v3/__http://pubs.acs.org/page/copyright/permissions_otherpub.html__;!!LQC6Cpwp!rbYd6EOnvQo8MvIJX0RnIEIBn89Ewtzk_9MNZiN3AWYkicdNEvARgHPyucJ3g4bHKmOiQqC3eqPFg3GMXp3UgoQXi1QuQ$

*If your graphic is reproduced or adapted from a source that is not published by the ACS, please submit the copyright permission form obtained from the other publisher as "Other Files for Editors Only". Please ensure that the licenses provide 1) "Type of use" as "reuse in journal/magazine", "Title of targeted journal" as "ACS Central Science", and 3) "Publisher" as "American Chemical Society". Most copyright permissions can be obtained from the publishers' websites. Please also make sure a permission statement is included in the caption, if you have not already done so, e.g., Reproduced or adapted with permission from [provide reference #]. Copyright [YEAR] [PUBLISHER].

*If you are using material that appeared in any ACS journal, and ACS owns copyright to the material, permission is NOT necessary. A simple acknowledgment is required, in this format, after inserting the appropriate information where the capitalized words appear: Reproduced from [PROVIDE REFERENCE #]. Copyright [YEAR] American Chemical Society.

*If you are using material that is covered under an Open Access license, please provide proper attribution, e.g., Reproduced from ref XX. Available under a CC-BY XX license. Copyright XXXX Author Name(s). For information about proper attribution, please see https://urldefense.com/v3/__https://wiki.creativecommons.org/wiki/Best_practices_for_attribution__;!!LQC6Cpwp!rbYd6EOnvQo8MvIJX0RnIEIBn89Ewtzk_9MNZiN3AWYkicdNEvARgHPyucJ3g4bHKmOiQqC3eqPFg3GMXp3Ugrtpr7lag$.

We thank the editor for this clarification. All structural panels (PDB IDs 1WDA and 3MZW) were generated de novo from publicly available coordinate data using molecular visualization software and do not reproduce or adapt previously published figures; appropriate citations to

the original structure publications have been added. The AlphaFold model is used under a CC-BY 4.0 license, and proper attribution has been included in the figure captions.

Supporting Information: If the manuscript is accompanied by any Supporting Information for Publication, a brief description of the supplementary material is required in the manuscript, before the reference list. The appropriate format is: Supporting Information. Brief statement in non-sentence format listing the contents of the material supplied as Supporting Information. Please list each supporting item individually.

*Examples of sufficient descriptions: "Supporting Information: 1H NMR spectra for all compounds" or "Additional experimental details, materials, and methods, including photographs of experimental setup."

*Examples of insufficient descriptions: "Supporting Information: Figures S1-S3" or "Additional figures as mentioned in the text."

We have added a description of Supplementary Information, including supplemental methods, figures, tables, and files, at the end of the text.

Reviewer(s)' Comments to Author:

Reviewer: 1

Recommendation: Major revisions required.

Comments:

This manuscript aims to understand the protein targets of extracellular protein arginine deiminase (PAD4) and the role of cell-surface citrullination in immunogenicity and cancer progression. The authors first demonstrate that treatment of EMT6 cells with PAD4 results in a change in morphology, generating more circular and less adherent cells. They then generated a ZHER2-PAD4 fusion protein where PAD4 is fused to the ZHER2 affibody, which would localize PAD4 at the surface of HER2-overexpressing cells. To identify protein targets of citrullination, the authors establish an HCD-product dependent-EThcD workflow. Negative control lysates (treated with DMSO or inactive PAD4) generated ~1000 citrullinated peptide identifications, whereas PAD4 or ZHER2-PAD4 samples afforded ~3000 citrullinated peptides. Lastly, EMT6 cells were citrullinated in vitro and used to immunize mice prior to implantation of EMT6 tumors. The resulting tumor take rate and the binding of mouse IgGs to citrullinated cells were measured.

We thank the Reviewer for appreciating the impact of this work and their careful and thoughtful comments.

Although the underlying premise and motivation for this study is of significant interest, the actual data that is presented falls short of providing significant insight into extracellular PAD function.

The focus of our work was to define what intracellular and extracellular proteins are citrullinated by PAD4 through an improved proteomic method, and the humoral response to them. We show that PAD induces changes in cell morphology which is novel and may help explain how it contributes to metastasis. We identify many new citrullinated proteins both in the extracellular (now emphasized) and intracellular space that provides an important new resource to better understand PAD biology.

The major concerns are outlined below:

1. The primary goal of this manuscript is to identify protein substrates of extracellular PADs, yet the main proteomic data provided is for cell lysates treated with exogenous PAD4. There are many studies out there that use a variety of methods to characterize PAD4 substrates in cell lysates and hundreds of citrullinated sites have already been identified. The main driving force for generating the HER2-PAD4 construct was to selectively look at extracellular (and not intracellular) PAD targets, but these data are not presented. The authors state that SKBR3 cells were treated with PAD4, membranes isolated and analyzed, yet these proteomic data are not presented nor discussed in any detail.

We thank the reviewer for their comments and agree that the SKBR3 surfaceome-targeting PAD4 data was underrepresented in our first submission. To address this, we have increased our depth of analysis on that dataset, adding significant figures of merit to uncover new PAD4 surfaceome substrates. We include a new Figure 5 in the main text of the manuscript which highlights the novel substrates on the SKBR3 cell surface.

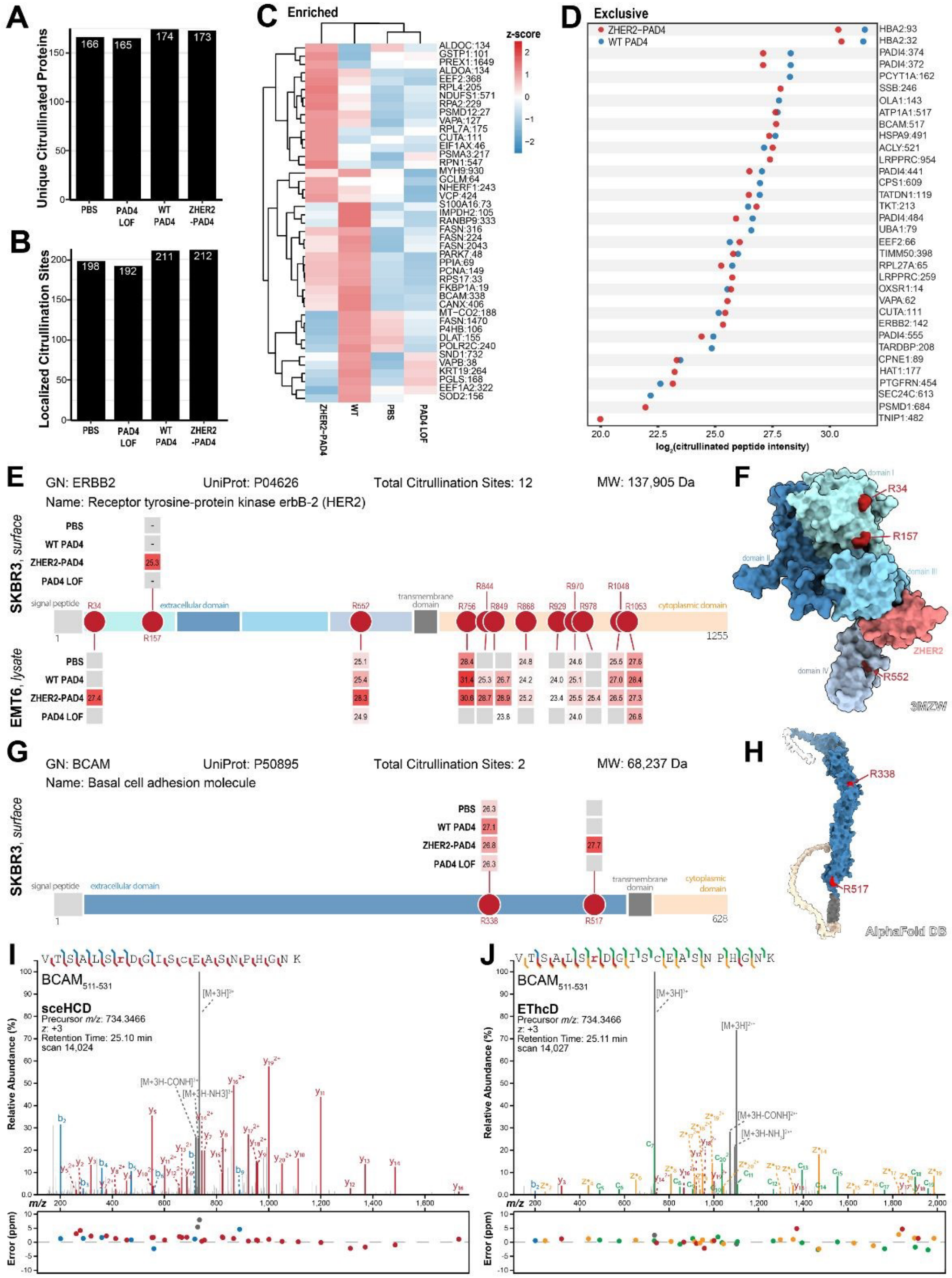

Fig 5. Deep proteomic profiling of intact SKBR3 cells to identify citrullinated substrates driving morphology and humoral responses. *Number of citrullinated (**A**) proteins, and (**B**) sites identified from the four treatment conditions. (**C**) Heatmap of enriched citrullination sites within active PAD4 conditions. Heat scores are represented as a z-score within each site. (**D**) Dot plot of exclusive localized citrullination sites not found in the PBS control. Modified peptide intensities were log2-transformed and plotted for wild-type or ZHER2-PAD4 conditions. (**E**) Mapping sites of hHER2 citrullination and their abundance from PAD4-treated intact SKBR3 (top) and EMT6 lysate (bottom). More sites of citrullination were identified in active PAD4 conditions and with higher abundance. In lysates both intracellular and extracellular sites of citrullination were observed; however, the differential signal for treatment with ZHER2-PAD4 vs PBS was higher for extracellular proteins. (**F**) Space filling rendering of the extracellular domain of hHER2 (showing the four sub-domains in different colors) bound to the ZHER2 binder in pink (PDB: 3MZW). (**G**) Mapping sites of BCAM*

*citrullination and their abundance from PAD4-treated intact SKBR3. (**G**) Space filling rendering of the AlphaFold DB structure of full-length BCAM with signal sequence in white, extracellular domain in blue, transmembrane domain in grey, cytoplasmic domain in orange, and localized citrullination sites in red. (**I**) sceHCD and (**J**) product-dependent EThcD MS/MS spectra for the BCAM peptide (amino acids 511-531) highlighting site-localization to R517 within the extracellular domain.*

2. More generally, the complete proteomic datasets were not provided making it difficult to judge the quality of the proteomic data.

While we intended to make the datasets as accessible as possible by uploading them to the MassIVE data repository prior to submission (PXD072496), this comment alerted us to the fact that the SKBR3 data were never uploaded which we are sorry for. We have remedied this and have now uploaded all proteomics data.

3. The authors identify ~1000 citrullination sites with over 50% occupancy in non-PAD treated lysates, which is unusual because endogenous levels of PADs are typically low in most cell lines and endogenous citrullination stoichiometry is typically well below 50% for any given site of citrullination. More explanation needs to be provided to explain this high background.

We thank the reviewer for this comment. The high stoichiometry we found was a feature seen whether PBS or inactive PAD4 (D350A) was added to lysate. We think represents the sensitivity of our method to detect endogenous substrates. Additionally, filtering was performed in revision which slightly reduced overall citrullinated site, peptide, and protein counts. WE now show for only peptides identified in both citrullinated and unmodified forms to qualify for Figure 4F. Due to the stochasticity of the LC-MS experiment, our earlier site occupancy values were inflated by citrullinated peptides that were not identified in their unmodified form. However, the stoichiometry for the endogenous unique citrullination sites are only one-third that of the active PAD4-treated lysate. While PAD4 expression may be low, these data reveal endogenous PAD substrates that are uniquely targeted for citrullination.

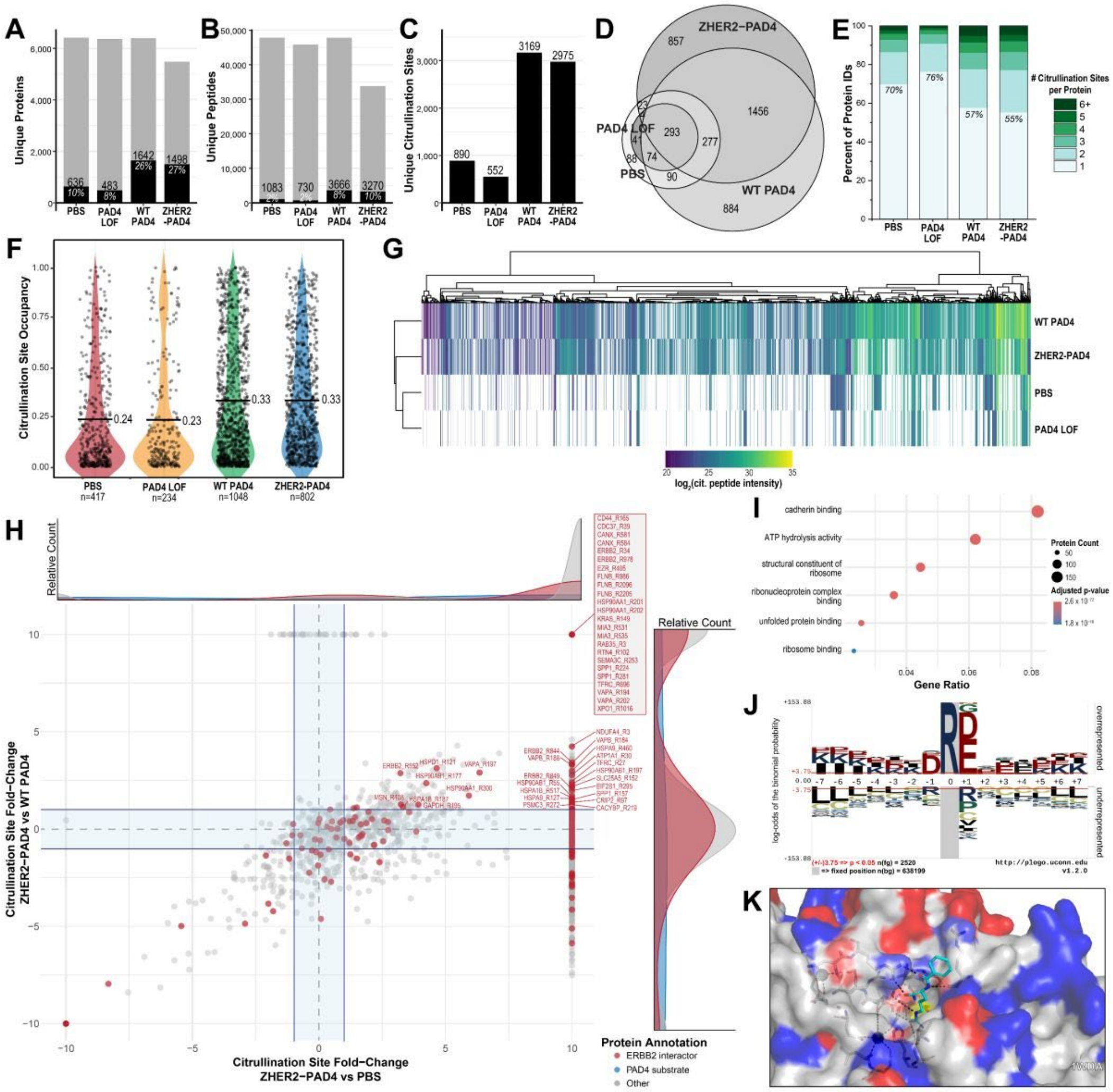

Fig 4. Deep proteomic profiling of cell lysate samples treated with PAD4 to identify unique, citrullinated substrates. *Number of citrullinated (**A**) proteins, (**B**) peptides, and (**C**) sites identified from the four treatment conditions along with all non-citrullinated sites. (**D**) Venn Diagram of unique citrullination sites across conditions. (**E**) Number of citrullination sites identified per protein. Note that treatment with active PAD4 produced 3 to 4 times more citrullinated peptides, proteins, and citrullination per protein than conditions without exogenously added PAD4 or PAD4 LOF. (**F**) Distribution of citrullination site occupancy for each treatment condition. For each site of highconfidence citrullination, the intensity of the citrullinated site was divided by the total site intensity, including multiple isoforms and charge states. Only sites detected in both modified and unmodified form are plotted to limit overestimation of site occupancy (i.e. sites only detected as citrullinated are omitted). Horizontal bars denote mean fraction of citrullination site occupancy. (**G**) Global heat map analysis of citrullinated peptides' log_2 transformed intensities from each experimental group. Missing values are shown as white. (**H**) Enrichment of citrullinated sites identified in ZHER2-PAD4 treated hHER2-ETM6 lysate plotted relative to endogenous citrullinated proteins identified in PBS treated (x-axis) or WT PAD4 (y-axis) treated lysates. Diagonal shows there is a general correlation for ZHER2-PAD4 activity whether compared to WT PAD4 or to PBS treatments. Sites within the center of the plot reflect mostly non-specific citrullination by PAD4. Many sites of citrullination shown at the far right, including HER2 sites, are found exclusively in the ZHER2-treated samples compared to PBS treatment. The upper right quadrant highlights sites that are heavily enriched in the ZHER2-PAD4 group relative to both background controls. (**I**) Gene ontology and molecular function analyses of proteins that were elevated in the ZHER2-PAD4 treatment of EMT6 lysates. (**J**) Consensus sequence Logo of citrullinated peptides. PAD4 shows strong preference for negatively charged residues surrounding site of citrullination and other charged residues on*

*the flanks. (**K**) Citrullinated peptide analogue bound at the active site of PAD4 enzyme (PDB: 1WDA). Several basic residues (blue) on PAD4 shown to interact with flanking negatively charged residues on citrullinated peptide.*

4. Details regarding how 'citrulline localization confidence' and 'site occupancy' estimations were calculated need to be provided to again better judge the quality of the proteomic data.

These descriptions are in Figure 3B and Figure 4F captions as well as the Methods. We have elaborated on these filtering parameters and calculations within the text to increase clarity.

"Citrullination site localization was filtered using a minimum localization probability threshold of 0.75, and only sites meeting this criterion were retained for downstream analysis. This means that there is at least a 75% likelihood that the 0.984 Da mass shifted Arginine is at the residue we are calling. This helps to reduce ambiguity with isobaric deamidation (+0.984 Da) on asparagine or glutamine residues. A localization threshold of 0.75 was used for citrullination. Peptide spectral matches (PSMs) corresponding to peptides terminating in a C-terminal citrulline residue were excluded, as citrullination neutralizes the positive charge of arginine and should result in a missed tryptic cleavage." (page 31, line 717)

5. The extensive discussion of HER2 citrullination sites seems extraneous to the main point of the manuscript which was to globally catalog cell-surface citrullination sites.

We thank the reviewer for this feedback. We have since increased discussion (and a new main figure (Figure 5)) for the surfaceome-specific PAD4 substrates in intact human SKBR3 cells and reframed discussion of the HER2 neighbors in EMT6 lysate. However, we think that citrullination of HER2 itself deserves necessary attribution, as that helps to validate the utility of our ZHER2-PAD4 reagent 1) as an active surface-targeting enzyme, 2) for enzyme promiscuity to non-reported substrates, and 3) to assess the distance at which PAD4 can function away from the ZHER2 binding interface of the HER2 extracellular domain. Indeed, ZHER2 only citrullinates arginine residues near the ZHER2 binding pocket of intact SKBR3 cells but can citrullinate arginine residues throughout HER2 for the EMT6 lysate experiments.

6. The measured differences in serum IgG binding to citrullinated cells (Fig 6 B, C and D) show a large degree of variability and low statistical significance. More data points would be helpful to more convincingly show that there is increased IgG binding.

We thank the reviewer for pointing out this variability in the serum IgG binding data. Each point represents one mouse, and five mice in each treatment group. Even though these experiments were conducted using a syngeneic mouse strain, it is not surprising to see biological variability among the treated group due to the larger values and natural variation in immunogenicity response to new antigens. We agree this is not a massive effect but it does pass statistical significance. Ideally we would like to increase our sample size, but these are also challenging and expensive experiments and we do not have resources to expand this part.

7. Given the importance of the tumor take rate experiments, these data should be presented within the main text and not in the SI.

We thank the reviewer for this helpful feedback. We have moved the tumor take rate experiments to the main text, adjoining them with Figure 6. See the new Figure 7 below:

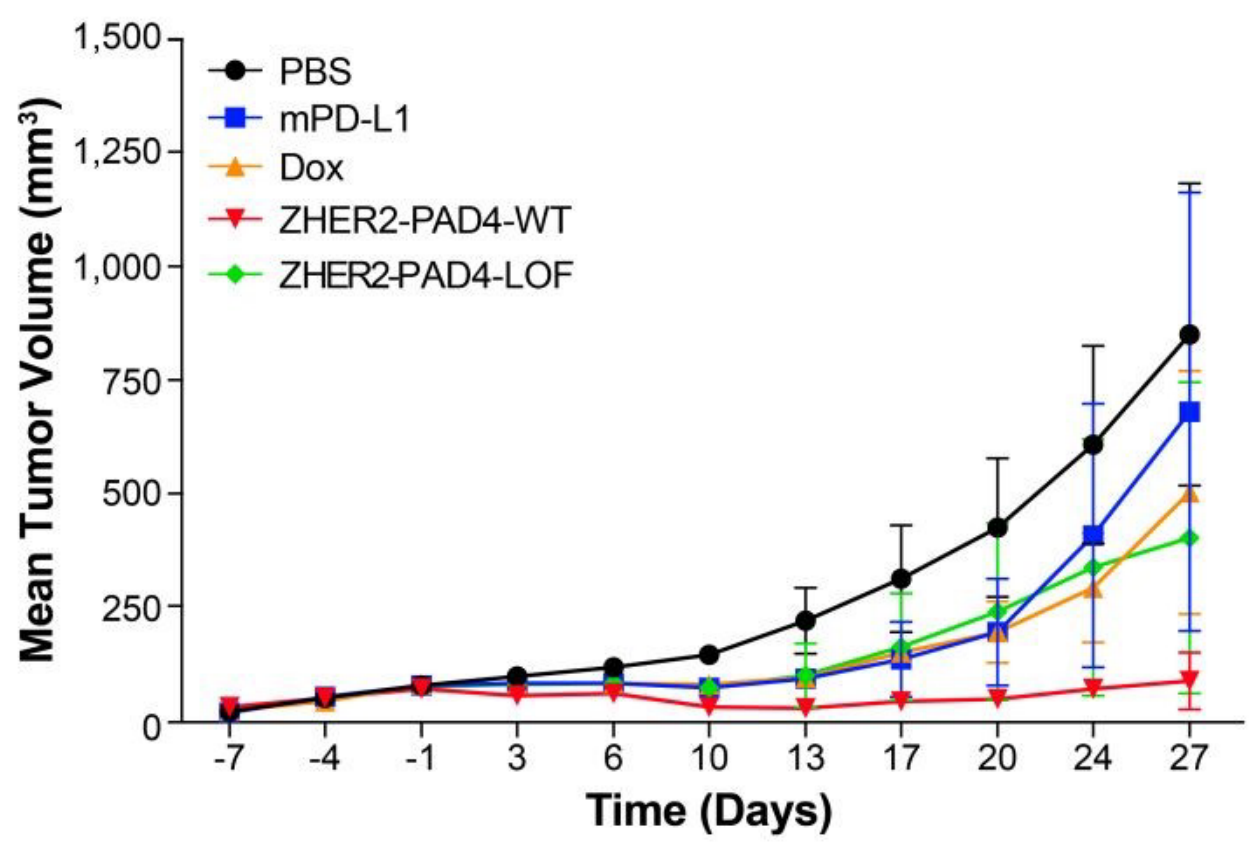

Fig 7. Tumor regression studies of mice implanted with EMT6 hHER2+ cells. *Mice subcutaneously implanted with EMT6 hER2+ cells were treated with PBS, mPD-L1, Dox, ZHER2-PAD4-WT, or ZHER2-PAD4-LOF. Mice treated with ZPAD4-WT had an observed delay in tumor growth compared to negative control and even positive control groups.*

Additional Questions:

Quality of experimental data, technical rigor: Moderate

Significance to chemistry researchers in this and related fields: Top 10%

Broad interest to other researchers: Top 10%

Novelty: Top 10%

Is this research study suitable for media coverage or a First Reactions (a News & Views piece in the journal)?: No

Reviewer: 2

Recommendation: Major revisions required.

Comments:

This manuscript presents a technically ambitious and multifaceted study examining extracellular PAD4 biology, engineering a HER2-targeted PAD4 fusion construct (ZHER2–PAD4),

and developing a neutralloss–triggered HCD pd EThcD proteomics workflow for citrullination site identification. Using these tools, the authors map global and HER2 proximal citrullination sites and further investigate whether PAD4mediated citrullination can act as a neoantigen source to stimulate humoral responses in a syngeneic mouse vaccination model. The work is innovative, and the proteomics pipeline represents a meaningful methodological advancement. The ZHER2–PAD4 fusion construct is well-designed and rigorously validated, and the authors successfully identify numerous endogenous and PAD4 dependent citrullination events. The manuscript addresses an important gap in understanding extracellular PAD4 biology and potential immunological consequences of citrullination.

However, significant conceptual, experimental, and interpretational issues remain. Several central claims—especially regarding proximity-based substrate identification, biological specificity of PAD4 activity, and conclusions drawn from the vaccination studies—are currently overstated or insufficiently supported. Logical gaps also arise from sudden transitions between major research components. Addressing these weaknesses would considerably strengthen the manuscript.

We thank the reviewer for their kind words and thoughtful analysis of the manuscript. We have addressed the concerns and comments below which we feel greatly improve the manuscript.

Major Concerns:

1. Conceptual inconsistency and logical gaps

A. Abrupt transitions between major

components The manuscript shifts rapidly

from:

1) Phenotypic effects of exogenous PAD4 →
2) Engineering ZHER2–PAD4 →
3) Lysate-based global citrullination mapping →
4) Intact cell–specific HER2 citrullination →
5) Vaccine immunology studies.

The narrative lacks connective tissue explaining why each transition is made and how they inform one another, which leads to two partially disconnected storylines: a targeted PAD4 engineering/proteomics story, and a general PAD4 based cancer vaccine story.

The authors should add clearer rationale linking each experimental module and clarify whether the primary scientific goal is mechanistic dissection, method development, or translational immunology.

We thank the Reviewer for this comment. To improve the continuity throughout the manuscript, we have moved and edited text to reduce the conceptual inconsistency and increase reader clarity. To bridge the main two elements (PAD4 engineering/proteomics toward PAD4-based cancer vaccine), we have elaborated on extracellular matrix and plasma membrane proteins which were citrullinated and likely drive morphological changes and possible humoral effects within cancer cells.

We also provide a new Supplemental Table (below) to summarize the intact SKBR3 citrullination sites via exogenously PAD4 across three classes of proteins which we believe may drive cell morphology and humoral response: 1) extracellular proteins, 2) membrane organization, 3) stress, oxidation, and
NETosis.

extracellular proteins		
**Gene**	**Protein Name**	**Site(s)**
ATP1A1	Basal cell adhesion molecule	517
BCAM	Basal cell adhesion molecule	338, 517
ERBB2	Receptor tyrosine-protein kinase erbB-2 (HER2)	142
PTGFRN	Prostaglandin F2 receptor negative regulator (CD315)	454
**membrane organization**		
**Gene**	**Protein Name**	**Site(s)**
HSPA9	Heat shock 70 kDa protein 9	491
MYH9	Myosin-9	930, 1652
SEC24C	Protein transport protein Sec24C	613
SLC25A4	ADP/ATP translocase 1	259
TIMM50	Mitochondrial import inner membrane translocase	398
VAPA	VAMP-associated protein A	58, 62, 127
VAPB	VAMP-associated protein B and C	38, 120
**stress / oxidative / NETosis**		
**Gene**	**Protein Name**	**Site(s)**
EEF2	Elongation Factor 2	66, 368, 580
FKBP1A	FK506-binding protein 1A	19
HBA2	Hemoglobin subunit alpha-2	32, 93
HNRNPK	Heterogeneous nuclear ribonucleoprotein K	148
IMPDH2	Inosine-5'-monophasphate dehydrogenase 2	105
OXSR1	Serine/threonine-protein inase OXSR1	14
PADI4	Protein-arginine deiminase type 4	372, 374, 441, 484, 555
PARK7	Protein deglycase DJ-1	48
PCNA	Proliferating cell nuclear antigen	91, 149
PPIA	Peptidyl-prolyl cis-trans isomerase A	37, 69
PSMD1	26S proteasome non-ATPase regulatory subunit 1	684
RANBP9	Ran-binding protein 9	333
SSB	Lupus La protein	246
TARDBP	TAR DNA-binding protein 43	208
TATDN1	TatD DNase domain-containing protein 1	119
TKT	Transketolase	213
TNIP1	TNFAIP3-interacting protein 1	482
UBA1	Ubiquitin-activating enzyme E1	79

2. Problems with the use and interpretation of lysate based proximity studies

A. Lysates cannot support claims of proximity-induced substrate selection

Lysate experiments destroy spatial protein organization, permitting promiscuous solution phase citrullination. Multiple reviewers correctly point out that the claimed “proximal” substrates in Fig. 4 are not meaningful in the context of induced proximity.

The authors should clarify that: lysate proteomics measures enzyme preferences, not spatial interactions; only intact-cell SKBR3 experiments support proximity regulated citrullination.

This is a good point and we agree that the initial submission over-emphasized HER2 interaction partners in EMT6 lysate experiments. In the revisions, we reduced mention of HER2 interactors for EMT6 data. While citrullination of proteins proximal to HER2 may still be enriched, we have chosen to focus more on such interactions in the SKBR3 surfaceome data. We include a new Figure 5 to this effect. Additionally, we have clarified the text to state:

"Annotating previously reported interacting partners of hHER2 reveals that many of the citrullination sites significantly upregulated in the ZHER2-PAD4 treatment are within neighborhoods or direct interactors of HER2. Importantly, lysate proteomics measures enzymes preferences while intact cell proteomics support proximity-induced citrullination. We repeated this study using whole SKBR3 cells that express high levels of HER2, treating again with PBS, WT PAD4, ZHER2-PAD4, or ZHER2-PAD4 LOF." (page 15, line 253)

B. Very high background citrullination in PBS and LOF controls

Detection of >1000 endogenous sites in controls raises concern about false positives or neutral-loss– triggered ID artifacts. The authors should validate a subset of endogenous hits with orthogonal methods or provide FDR benchmarking.

We thank the reviewer for this comment. We agree that identification of a large number of endogenous citrullination sites warrants careful control of false positives. In our workflow, multiple orthogonal filters were applied to ensure high-confidence site assignment. Specifically, peptide and protein identifications were controlled at 1% FDR at the PSM, peptide, and protein levels using FragPipe. Citrullination sites were further filtered using a localization probability threshold of ≥0.75 to ensure confident assignment to arginine residues and to distinguish citrullination from isobaric deamidation events on asparagine or glutamine residues.

In addition, product-dependent EThcD acquisition was employed, requiring the presence of the diagnostic neutral loss of isocyanic acid (43.0058 Da) in the triggering HCD scan prior to EThcD fragmentation. This provides orthogonal confirmation of citrullination and reduces the likelihood of false-positive identifications arising from stochastic fragmentation or database matching artifacts.

In revision, we have re-filtered the data in this way and now report fewer than 1,000 localized sites in the PBS and ZHER2-PAD4 LOF treatments of EMT6 lysate.

We also note that endogenous citrullination has been reported at appreciable levels in cellular systems, particularly under conditions of stress or PAD4 activity, and the observed sites are enriched in biologically plausible protein classes (e.g., cytosolic, nuclear, and membrane-

associated proteins), supporting the validity of the dataset. Please also see above where we addressed a similar comment from Reviewer #1.

C. Missing essential negative control

PAD4 LOF was used instead of ZHER2–PAD4 LOF, despite the latter being available and more appropriate.

We indeed used a ZHER2-PAD4 D350A rather than a PAD4 D350A in these experiments. We have clarified the text and Figures to that effect.

3. Inconsistencies in PAD4 binding and activity across assays

Several discrepancies must be clarified:

- WT PAD4 binds HER2+ cells by flow cytometry (Fig. 2C), yet shows nearly no citrullination by Western (Fig. 2E).
- SKBR3 intact-cell MS shows no WT PAD4 citrullination of HER2, contradicting earlier data.

The authors should discuss assay sensitivity, kinetics, and potential differences between biochemical and intact cell environments.

We thank the reviewer for these important clarifications. Indeed, flow cytometry can be more sensitive than western blotting and sensitivity limitations of WB and background signal of flow cytometry likely explain the inconsistency between Fig 2C and 2E. We note that the flow cytometry data reveal marginal binding of WT PAD4 to HER2+ cells relative to vehicle and the ZHER2-PAD4 treatment. Indeed, average signal for WT PAD4 is ~300x lower than for ZHER2-PAD4. See Fig 2C, below:

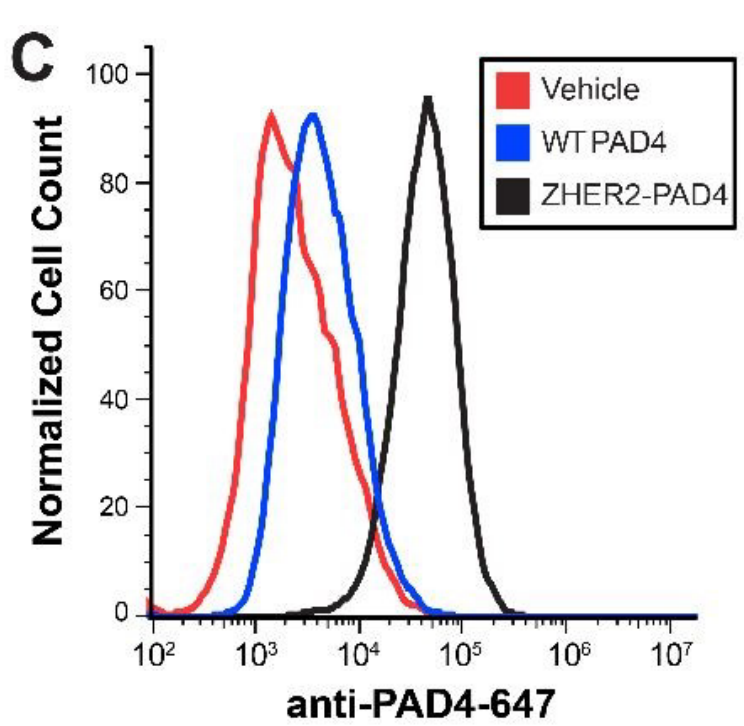

4. Issues with Figure 1 and biological interpretations

- No images are provided to support quantified morphology metrics.
- N values for all panels (A–D) are missing.

- Changing cell line (EMT6 → Patu8988t) for migration studies lacks justification and biological rationale.

Recommendation: The authors should add representative images, replicate numbers, reasoning for cellline choice, and discussion connecting morphology to specific citrullinated substrates.

We have added cell images to Figure 1 corresponding to cellular morphology changes induces by PAD4. We have added N values to all panels. Additionally, we provided a sentence of clarity as to why Patu8988t cells were used for cell migration studies:

"*Migration assays were performed in Patu8988t cells due to their robust intrinsic migratory capacity and low levels of cellular migration when untreated, allowing sensitive detection of changes in cell motility. EMT6 cells exhibit limited migration in vitro, making them less suitable for quantifying cell-intrinsic migratory phenotypes. Patu8988t cells were plated in serum-free media and we monitored migration into complete media, plus or minus PAD4 or variants*." (page 6, line 110)

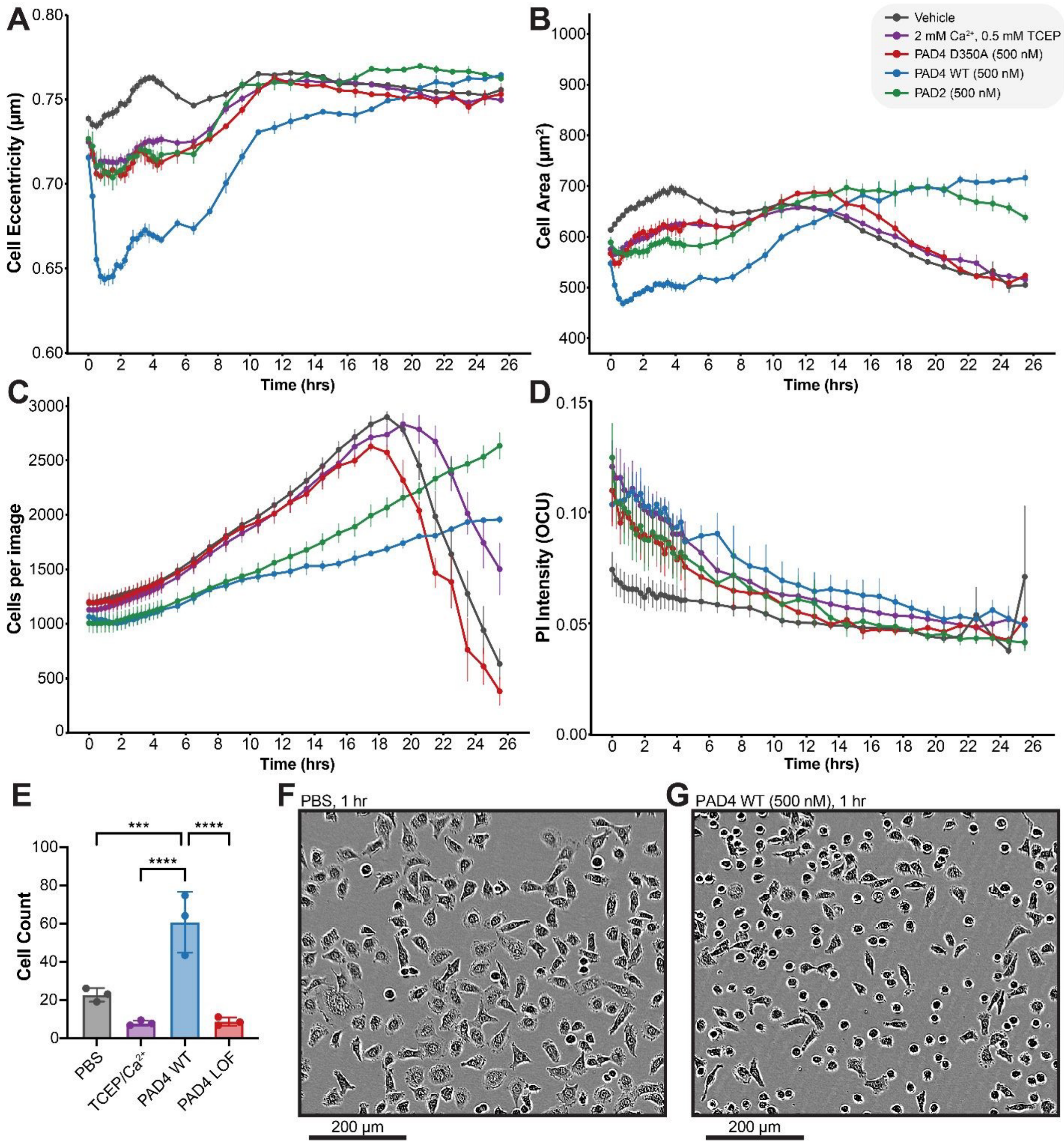

Fig. 1. Treatment of EMT6 cells with active PAD4 versus a loss-of function variant differentially changes cell morphology, growth rates, viability, and migration. *Cells were treated for 24 hours and different parameters measured by incucyte. (**A**) Cellular eccentricity defined as a less spherical cell shape, (**B**) cell area, (**C**) cell count, and (**D**) cell viability measured by propidium iodine stain. (**E**) Transwell migration of Patu8988t cells across a porous membrane. PAD4 treated cells have an increase propensity for migration as compared to untreated, vehicle, or PAD4 LOF treated cells. Bars represent standard error across three biological replicates. Representative brightfield images of (**F**) PBS or (**G**) 500 nM PAD4 WT treated EMT6 cells 1 hr after treatment.*

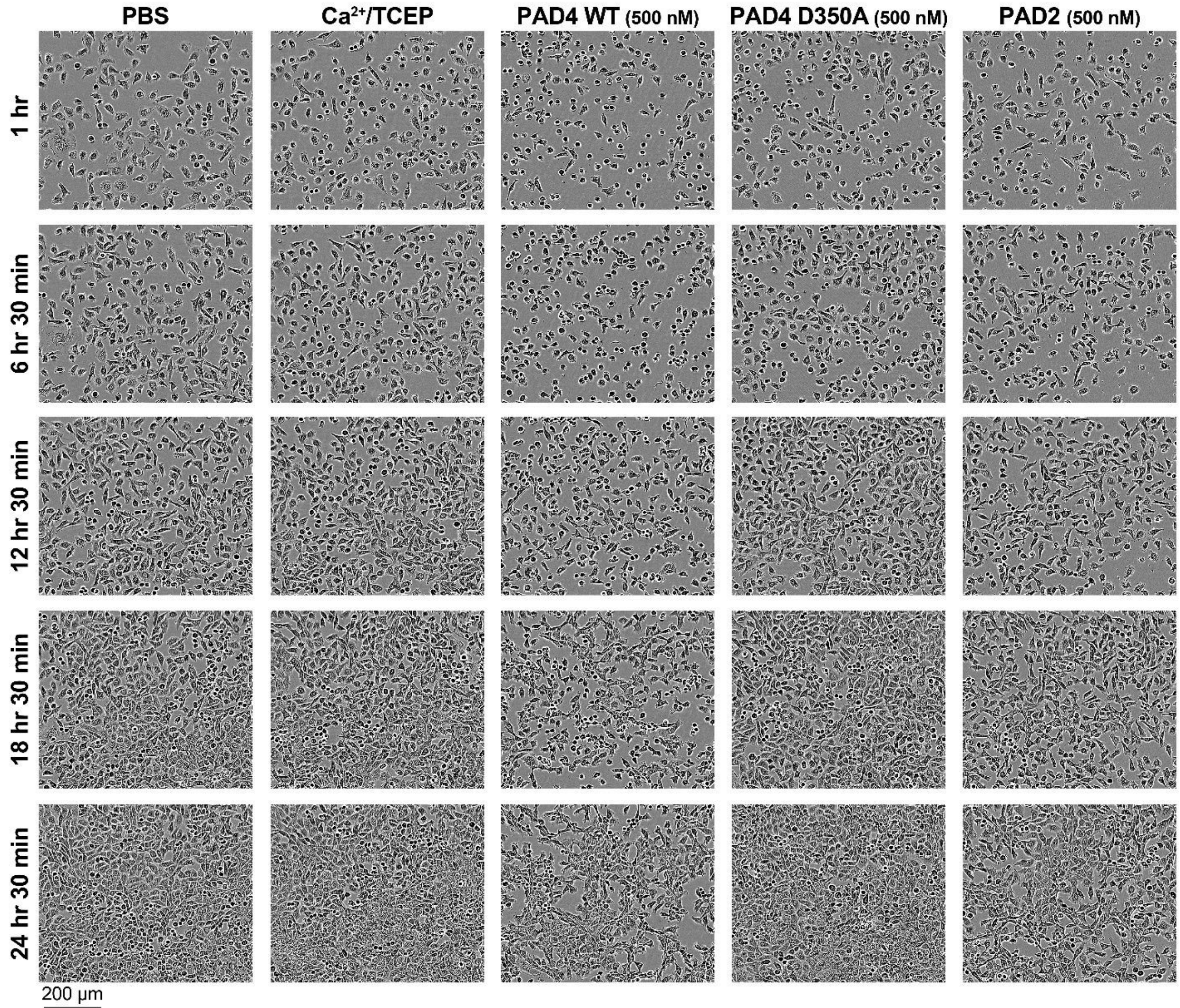

Fig. S7. Representative incucyte images of EMT6 cells treated with PAD4 or controls. *Cells were treated for 24 hours and different parameters measured by incucyte. Brightfield images are shown for each of the five conditions 1 hr through 24.5 hr following treatment.*

5. Mass spectrometry workflow: missing performance metrics

- No assessment of neutral loss triggering efficiency.
- No comparison with standard ETD workflows in complex samples.
- No discussion of potential false negatives.

The authors should include benchmarking or limitations discussion.

We include an assessment of the neutral loss triggering efficiency as Supplemental Figure S4 (*below*). We elected not to compare our HCD-pd-EThcD method with standard ETD proteomics as the duty cycle of ETD is so much slower than HCD that many fewer precursor ions could be

selected, fragmented, and identified. Use of ETD likely wouldn't have conferred any benefit over EThcD. Many studies have shown that EThcD aids in generation of c/z ion pairs and b/y ion pairs relative to ETD alone. While the comparison of standard ETD to our HCD-pd-EThcD method would've made our strategy look better, we elected to simply use the most efficient method while also gaining citrulline confidence from isocyanic acid loss.

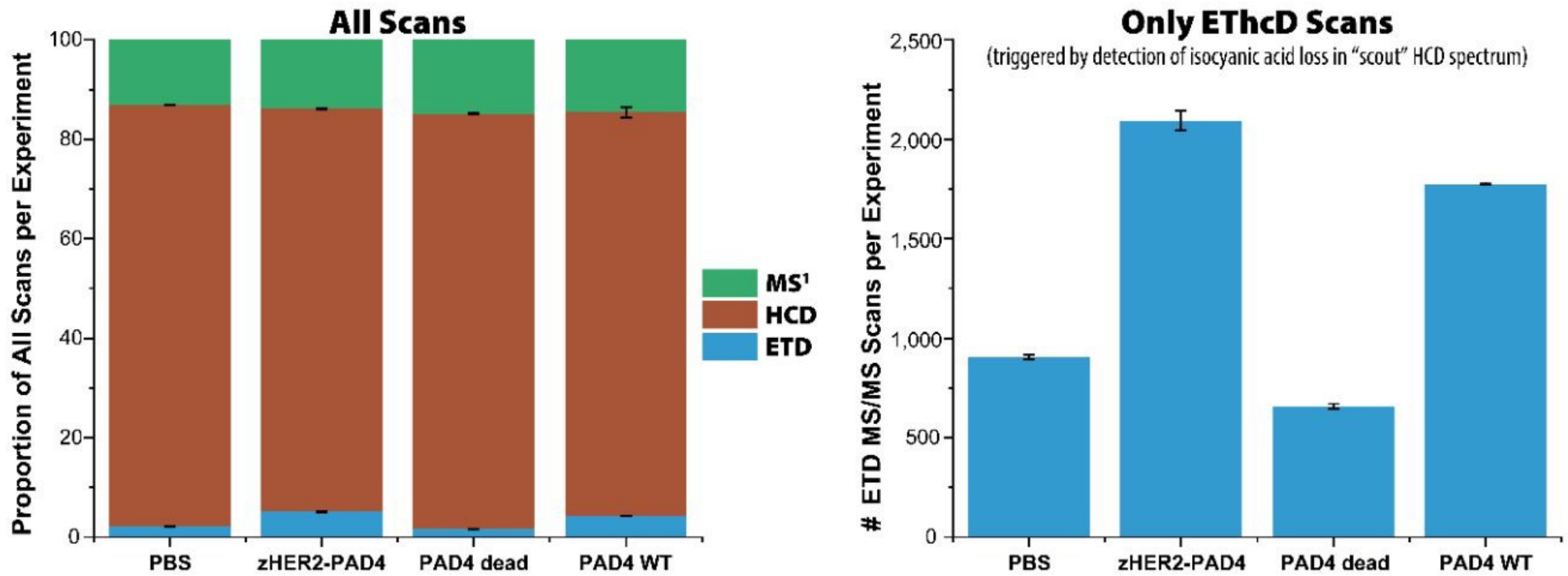

Fig S4. Breakdown of scan types within HCD-pd-EThcD intelligent data acquisition for citrullination profiling of EMT6 lysate. *Triggering an EThcD scan necessitates longer scan times and slower duty cycles. Performing a quick HCD scan will release a labile isocyanate neutral loss from citrulline residues, an event that produces a unique diagnostic ion. Matching this mass shift (-43 Da) from the intact precursor (±10 ppm) in the scout HCD scan triggers a sequence informative and citrulline localizing EThcD scan. While all conditions produced some level of EThcD scans, the relative number of EThcD scans was highest for active PAD4 (WT and zHER2 targeted). The zHER2-PAD4 treatment of EMT6 lysate generated the highest number of triggered scans, over double the number of EThcD scans as the PBS and zHER2-PAD4 LOF treatments. This indicates high enrichment of citrulline-containing peptides within the zHER2-PAD4 sample.*

6. HER2 citrullination analyses: conflicting statements and missing data

- Authors state only ZHER2–PAD4 produces extracellular citrullination on HER2, but text and figures appear partially inconsistent.

We have worked to eliminate inconsistencies throughout the text.

- Quantitative summary of SKBR3 citrullinated peptide counts is missing.

Our new Figure 5 (shown above) includes a full panel of SKBR3 figures of merit, including citrullinated proteins and localized citrullination sites for PBS, ZHER2-PAD4 LOF, WT PAD4, and ZHER2-PAD4 treatment.

7. Immunization study design issues

A. Missing pre challenge serum timepoint

Without Week 0 serology, it is impossible to isolate vaccine induced immunity from tumor induced recall responses.

B. Lack of negative target controls The authors claim antibodies target citrullinated epitopes, but binding to non citrullinated cells is not shown. C. Lack of T-cell functional readouts

The manuscript claims immunological relevance but provides only IgG binding data.

The authors should either temper claims or include additional functional immunology data.

We thank the reviewer for pointing out concerns with our immunization study design. We did not collect week 0 serology, however we did check for serum binding to non-citrullinated cells across all experimental groups (Fig. 6B). In groups treated with citrullinated cells, we saw increased serum binding to *in vitro* citrullinated cells but not to non-citrullinated cells.

We acknowledge that this is a valid concern and have tempered the claims that we are not measuring Tcell responses but rather humoral, IgG responses in response to varied treatment. We understand that humoral responses and ACPA generation are inherently tied to T-cell responses, but our work set out to investigate specifically ACPA responses. Our lack of understanding of the T-cell response may be the reason we don't observe an anti-tumor growth effect. However, we believe that one of the reasons for that is that EMT6 tumors are very aggressive and cell growth may out-pace slower full immunogenicity effects.

Minor Issues and Required Corrections

(Condensed for clarity—full details reflected directly from reviewers)

- Ensure consistent terminology (e.g., EMT6 vs EMT 6).

We thank the reviewer for this feedback. We have edited the text to remove inconsistencies and have aligned terminology, especially in regard to the EMT6 cell line.

- Provide gating strategies for all flow cytometry experiments.

Flow cytometry gating strategies are now included in the Methods section. (page 25, line 490-492)

- Improve clarity of figure labels, especially in Fig. 5.

We have increased the clarity of figure captions.

- Cite prior publications where needed (e.g., histone H3 citrullination sites).

We include several references to earlier PAD4 and histone H3 literature including the following: *"Previous research identified four additional citrullination sites on H3 using a partial proteolysis strategy to cover Arg-dense regions.[56]" (page 11, line 177)*

"Previous studies using histone H.3 peptide derivatives have shown PAD4 to be a promiscuous enzyme with broad substrate specificity centered around the Arg.[59]" (page 15, line 240)

- Add methodological details on data normalization, missing value handling, heatmap scaling.

We have added methodological details clarifying that equal peptide mass was injected for each run, no global normalization or match-between-runs was applied, and missing values were not imputed. We also clarified the heatmap scaling used in each panel (log2 intensity and/or row-wise z-score) and how missing values were displayed. *(page 29, line 531)*

Overall, this manuscript has high potential for impact in chemical biology, proteomics, and immunology. The engineered ZHER2–PAD4 and the HCD pd EThcD workflow are valuable contributions that many groups will adopt. The proteomic dataset will serve as a foundational resource for the citrullination community. However, publication in ACS Central Science requires substantial conceptual clarification and strengthening of experimental interpretations. Addressing the issues above—especially proximity claims, methodological limitations, inconsistencies across assays, and vaccination study design—will greatly enhance rigor and coherence.

We thank the reviewer for this positive feedback and constructive comments that we feel have led to a more robust and clearer manuscript. We have toned down the proximity claims involving lysate datasets and added clear notes of methodological limitations introducing what appear to be inconsistencies (e.g. between western blot data and LC-MS/MS data.

Additional Questions:

Quality of experimental data, technical rigor: Moderate

Significance to chemistry researchers in this and related fields: Top 10%

Broad interest to other researchers: Top 10%

Novelty: Top 10%

Is this research study suitable for media coverage or a First Reactions (a News & Views piece in the journal)?: No

oc-2026-00032r.R2

Name: Peer Review Information for "Cellular consequences, citrullination substrates, and antigenicity of targeting PAD4 to cell surfaces"

Second Round of Reviewer Comments

Reviewer: 1

Comments to the Author

The authors have addressed some of the previous reviewer concerns, and have now included a figure focused on the SKBR3 cell-surface citrullination data. However, as noted in prior reviews, the SKBR3 cell-surface citrullination dataset (and not the lysate data) is critical to the central goal of the manuscript, which is to map extracellular citrullination sites using a HER2-targeted PAD4, yet, this dataset is of insufficient quality to support publication for the following reasons:

1. There is no difference in the number of proteins/peptides citrullinated in the active HER2-PAD4/PAD4 versus inactive PAD4/PBS treatments. If the goal is to identify PAD4-mediated cell-surface citrullination events, then there needs to be a significant increase in the identifications from the PAD4 treated cells to support that the exogenous PAD4 is actively citrullinated cell-surface proteins.

2. Most of the high abundant citrullination sites identified in the HER2-PAD4 treatment are not extracellular proteins, again raising questions regarding whether these are true HER2-PAD4-mediated extracellular citrullination events. The data need to be parsed to clearly show a list of extracellular citrullination sites identified.

3. There are few HER2 interactors identified. Again, the expected outcome is that the HER2-PAD4 construct should show increased citrullination of extracellular regions of HER2 and its interacting partners - the data provided, short of a single site on HER2, does not support this hypothesis.

4. An excel file containing a list of all the identified peptides should be included, with sites of citrullination, PSMs, abundance etc. as supplementary data for the manuscript. This should be provided for the histone citrullination data, the cell lysate data and the intact cell analyses. This would enable the reader to get further insight into the proteins and sites identified in each of these experiments.

Given the importance of the SKBR3 intact-cell labeling experiments, and the inconsistencies in this important dataset noted above, this manuscript falls short of the

main goal of identifying extracellular sites of citrullination. I therefore cannot recommend publication of this manuscript in its current form.

Reviewer: 2

Comments to the Author

The authors have made good efforts to address most of my previous concerns. The revised manuscript has greatly improved clarity and meets the high standard of ACS Central Science for publication.

Author's Response to Peer Review Comments:

Dear Editor,

Thank you for the opportunity to revise our manuscript a second time. We are delighted that Reviewer #2 felt the paper was appropriately revised and appreciate the additional comments from Reviewer #1. We have done our best to address these below with additional analysis requested and through editorial changes.

Reviewer: 1

Recommendation: Major revisions required.

Comments:
The authors have addressed some of the previous reviewer concerns, and have now included a figure focused on the SKBR3 cell-surface citrullination data. However, as noted in prior reviews, the SKBR3 cell-surface citrullination dataset (and not the lysate data) is critical to the central goal of the manuscript, which is to map extracellular citrullination sites using a HER2-targeted PAD4, yet, this dataset is of insufficient quality to support publication for the following reasons: 1. There is no difference in the number of proteins/peptides citrullinated in the active HER2PAD4/PAD4 versus inactive PAD4/PBS treatments. If the goal is to identify PAD4-mediated cellsurface citrullination events, then there needs to be a significant increase in the identifications from the PAD4 treated cells to support that the exogenous PAD4 is actively citrullinated cellsurface proteins.

We thank the reviewer for this thoughtful comment. It was our intent to test the hypothesis that

membrane targeting by fusing PAD4 to HER2 would enable higher levels of surface citrullination. To better demonstrate the enrichment from targeting PAD4 to the membrane, we added a new **Figure 5** highlighting citrullination sites that are enriched in or unique to the active zHER2-PAD4 and WT PAD4 treatments compared with the catalytically inactive D350A PAD4 and PBS controls.

While the total number of citrullinated peptides identified across treatment groups is only modestly elevated with surface-targeted PAD4, we do not believe this metric alone should be used solely to judge the publication of this work nor does it reflect the ability of exogenous PAD4 to generate extracellular citrullination events. Several biological and technical factors contribute to this observation.

First, plasma membrane proteins comprise only about 1% of the total cellular proteome. Without dedicated cell-surface enrichment, extracellular citrullinated peptides are substantially underrepresented relative to the abundant intracellular proteome. Thus, differences in surface citrullination are expected to have only a modest effect on the total number of identified citrullinated peptides.

Second, endogenous PAD enzymes are known to citrullinate numerous intracellular substrates, and additional citrullination may occur after cell lysis during sample preparation. These endogenous events contribute to the background citrullination detected across all treatment groups, including the D350A and PBS controls.

Third, the ZHER2-PAD4 construct will only be able to act on cell surface substrates which present accessible arginines and meet the structural constraints defined by the ZHER2 binding epitope of HER2, the short G4S linker, and HER2 proximal proteins.

In addition, SKBR3 cells express exceptionally high levels of HER2. This high receptor density likely constrains both WT PAD4 and zHER2-PAD4 at the cell surface, reducing the overall differences between these treatment groups while still permitting identification of substrates uniquely modified by the HER2-targeted construct.

Importantly, despite these sources of background, the zHER2-PAD4 treatment uniquely identified extracellular citrullination sites on HER2 and BCAM. Both proteins are known to reside within the HER2 membrane neighborhood (approximately 10 nm), consistent with proximitydependent substrate modification by the targeted enzyme (*Bartholow et al., 2024; MacMillan et al., 2025; Maza et al., 2026*). These findings provide direct evidence that targeted PAD4 can generate extracellular citrullination events on biologically relevant cell-surface substrates.

Finally, one of the major contributions of this work is the development of a robust mass spectrometry workflow capable of identifying PAD4-mediated citrullination events in both

defined *in vitro* systems and complex biological samples, via endogenous or exogenous PAD4. This important new methodology will be important for the field of citrullination as a major posttranslational modification. We believe the selective identification of extracellular protein domain citrullination, together with the methodological advances presented here, supports the central conclusions of the manuscript.

Please see **Figure 5** below:

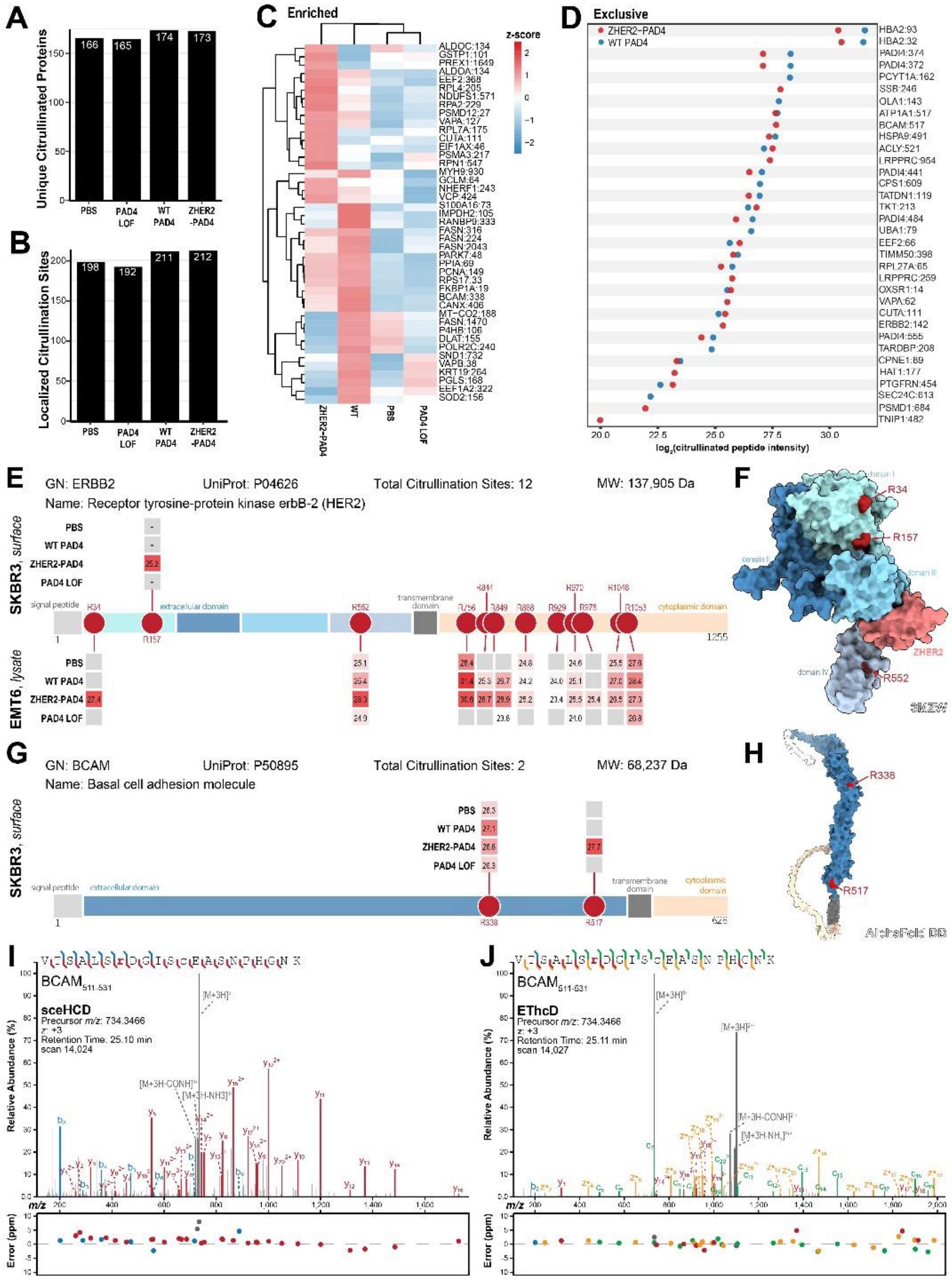

Fig 5. Deep proteomic profiling of intact SKBR3 cells to identify citrullinated substrates driving morphology and humoral responses. *Number of citrullinated (**A**) proteins, and (**B**) sites identified from the four treatment conditions. (**C**) Heatmap of enriched citrullination sites within active PAD4 conditions. Heat scores are represented as a z-score within each site. (**D**) Dot plot of exclusive localized citrullination sites not found in the PBS control. Modified peptide intensities were log2-transformed and plotted for wild-type or ZHER2-PAD4 conditions. (**E**) Mapping sites of hHER2 citrullination and their abundance from PAD4-treated intact SKBR3 (top) and EMT6 lysate (bottom). More sites of citrullination were identified in active PAD4 conditions and with higher abundance. In lysates both intracellular and extracellular sites of citrullination were observed; however, the differential signal for treatment with ZHER2-PAD4 vs PBS was higher for extracellular proteins. (**F**) Space filling rendering of the extracellular domain of hHER2 (showing the four sub-domains in different colors) bound to the ZHER2 binder in pink (PDB: 3MZW). (**G**) Mapping sites of BCAM citrullination and their abundance from PAD4-treated intact SKBR3. (**G**) Space filling rendering of the AlphaFold DB structure of full-length BCAM with signal sequence in white, extracellular domain in blue, transmembrane domain in grey, cytoplasmic domain in orange, and localized citrullination sites in red. (**I**) sceHCD and (**J**) product-dependent*

EThcD MS/MS spectra for the BCAM peptide (amino acids 511-531) highlighting site-localization to R517 within the extracellular domain.

2. Most of the high abundant citrullination sites identified in the HER2-PAD4 treatment are not extracellular proteins, again raising questions regarding whether these are true HER2-PAD4mediated extracellular citrullination events. The data need to be parsed to clearly show a list of extracellular citrullination sites identified.

We thank the reviewer for this suggestion. We agree that clearly distinguishing extracellular from intracellular substrates strengthens the presentation of the cell-surface citrullination data.

To address this point, we have added a new **Table 1** to the main text that specifically summarizes plasma membrane, cell surface-associated, and secreted proteins identified as citrullinated following treatment with zHER2-PAD4 and/or WT PAD4. This table highlights extracellular substrates independently of the broader dataset, allowing readers to readily evaluate the cell-surface citrullination events generated by exogenous PAD4.

The presence of intracellular citrullinated proteins in the complete dataset is not unexpected. Numerous cytosolic proteins are established endogenous PAD substrates, and these contribute to the background citrullination detected by mass spectrometry. Our objective was therefore not to exclude intracellular citrullination, but rather to identify additional extracellular substrates generated by exogenous PAD4. The fact that many of these are not surface proteins can derive from two effects often seen in surfaceomics experiments: 1) the surfaceome is about 1% as abundant and the cytosolome, introducing an analytical challenge and justification for our new proteomic method for detecting citrullination. Cell lysis or apoptosis which is common in cell culture, will present abundant cytosolic proteins to be citrullinated. 2) it has now been established that cytosolic proteins from lysis can associate with the membrane, often through electrostatic interactions with heparin sulfate (T Slezak and AA Kossiakoff, PNAS 2026).

Importantly, the newly added **Table 1** demonstrates that exogenous PAD4 citrullinates multiple extracellular and membrane-associated proteins, including HER2 (ERBB2), BCAM, SLC44A2, ATP1A1, PTGFRN (CD315), transferrin receptor (TFRC), and NHERF1. Notably, extracellular citrullination of HER2 and BCAM was observed uniquely following zHER2-PAD4 treatment, consistent with localized substrate modification by the HER2-targeted enzyme. These findings support our conclusion that targeted PAD4 can generate extracellular citrullination on biologically relevant cell-surface proteins.

We have added a comment within the manuscript to this effect:

“The catalogued citrullination sites include numerous abundant intracellular proteins, reflecting the predominance of intracellular proteins in whole-cell proteomic analyses

together with endogenous PAD activity. To highlight extracellular substrates identified following exogenous PAD4 treatment, we compiled plasma membrane, cell surface-associated, and secreted proteins detected as citrullinated in the intact-cell and lysate datasets (Table 1). These proteins include HER2, BCAM, SLC44A2, ATP1A1, PTGFRN, TFRC, and NHERF1." (page 18, line 367).

Gene	Protein Name	Site(s)
ATP1A1	Na^+/K^+-transporting ATPase subunit alpha-1	30[e], 61[e], 74[e], 198[e], 227[e], 517[i], 658[e]
BCAM	Basal cell adhesion molecule	338[i], 517[i]
CD44	CD44 antigen	165[e]
CLIC3	Chloride intracellular channel protein 3	168[i]
CTTN	Src substrate cortactin	38[e], 59[e], 94[e], 97[e], 131[e], 242[e]
ERBB2	Receptor tyrosine-protein kinase erbB-2 (HER2)	34[e], 142[i], 552[e], 756[e], 844[e], 849[e], 868[e], 929[e], 970[e], 978[e], 1048[e], 1053[e]
NHERF1	Na^+/H^+ exchange regulatory cofactor NHE-RF1	58[i], 243[i]
NISCH	Nischarin	945[e], 1029[e]
PTGFRN	Prostaglandin F2 receptor negative regulator (CD315)	454[i]
SLC44A2	Choline transporter-like protein 2	70[i]
TFRC	Transferrin receptor	27[e], 123[e], 527[e], 696[e]

Table 1. *Extracellular and plasma membrane-associated proteins identified as citrullinated following exogenous PAD4 treatment. i: intact SKBR3 cells; e: EMT6 cell lysate.*

3. There are few HER2 interactors identified. Again, the expected outcome is that the HER2PAD4 construct should show increased citrullination of extracellular regions of HER2 and its interacting partners - the data provided, short of a single site on HER2, does not support this hypothesis.

We thank the reviewer for this comment. We respectfully note that **Figure 5D** and **5E** demonstrate distinct citrullination profiles between WT PAD4 and ZHER2-PAD4 treatment and our controls. Moreover, HER2 and BCAM, both cell surface proteins associated with the HER2 membrane neighborhood, were identified as uniquely citrullinated following ZHER2-PAD4 treatment.

While we agree that additional HER2-associated proteins were hoped to be seen in the dataset. Again, we were testing a hypothesis, and one we believe had merit. We did not necessarily expect all HER2 neighbors to become citrullinated. Unlike proximity labeling enzymes that generate reactive and diffusive probes, PAD4 requires productive enzyme-substrate interactions with solvent-accessible arginine residues while tethered to HER2. The short G4S linker used in the ZHER2-PAD4 construct, together with the orientation of HER2 on the cell surface and the spatial arrangement of neighboring membrane proteins, likely restricts the subset of proteins and arginine residues accessible for catalysis. Consequently, the absence of citrullination on a known HER2 neighbor should not be interpreted as evidence that the protein was not proximal to HER2.

To clarify this point, we have added text to the Discussion noting that linker length, enzyme orientation, and substrate accessibility are likely determinants of the extracellular citrullination landscape generated by targeted PAD4:

"Unlike proximity labeling approaches that modify proteins within a defined diffusion radius of reactive biotinylated probes, targeted PAD4 requires productive catalytic engagement with accessible arginine residues. Consequently, the extracellular citrullination landscape is restricted by linker length, enzyme orientation, and substrate accessibility in addition to protein proximity." (page 23, line 501)

4. An excel file containing a list of all the identified peptides should be included, with sites of citrullination, PSMs, abundance etc. as supplementary data for the manuscript. This should be provided for the histone citrullination data, the cell lysate data and the intact cell analyses. This would enable the reader to get further insight into the proteins and sites identified in each of these experiments.

We thank the reviewer for this suggestion and agree that providing the identified citrullinated peptides in an easily accessible format improves the utility of the dataset. We have added supplementary Excel tables containing all identified citrullinated peptides from the histone H3 *in vitro* experiments, the whole-cell lysate analyses, and the intact cell experiments. These tables include the identified peptides, sites of citrullination, peptide-spectrum matches (PSMs), abundance measurements, and associated protein annotations. The complete raw mass spectrometry data remain available through the MassIVE and PRIDE repositories (MSV000100349).

Given the importance of the SKBR3 intact-cell labeling experiments, and the inconsistencies in this important dataset noted above, this manuscript falls short of the main goal of identifying extracellular sites of citrullination. I therefore cannot recommend publication of this manuscript in its current form.

We thank the reviewer for their additional comments and believe the additional revisions improves the overall clarity and quality of the manuscript. Specifically, we have: 1) added new analyses highlighting citrullination sites enriched in or unique to active PAD4 treatments (**Figure 5**), 2) included a new Table 1 summarizing extracellular, plasma membrane-associated, and secreted citrullinated proteins, 3) expanded the Discussion to clarify the biological and analytical factors influencing the intact-cell dataset, and 4) provided complete citrullination site datasets as supplementary Excel files.

While the intact-cell proteomics analysis provides an important component of the study, it represents one aspect of a broader investigation into the biological consequences of targeted PAD4. Together with the observed effects on cell morphology, cell growth, and humoral immune responses, the proteomic analyses support the conclusion that cell surface-targeted PAD4 generates biologically relevant extracellular citrullination and alters tumor cell phenotype and antigenicity. We hope these revisions adequately address the concerns raised. We look forward to hear back regarding next steps.

Additional Questions:

Quality of experimental data, technical rigor: Moderate

Significance to chemistry researchers in this and related fields: Top 10%

Broad interest to other researchers: Top 10%

Novelty: Top 10%

Reviewer: 2

Recommendation: Publish in ACS Central Science without change.

Comments:

The authors have made good efforts to address most of my previous concerns. The revised manuscript has greatly improved clarity and meets the high standard of ACS Central Science for publication.

Additional Questions:

Quality of experimental data, technical rigor: Top 10%

Significance to chemistry researchers in this and related fields: Top 10%

Broad interest to other researchers: Top 10%

Novelty: Top 10%
